# Membrane-Interacting Antifungal Peptides

**DOI:** 10.3389/fcell.2021.649875

**Published:** 2021-04-12

**Authors:** Caroline Struyfs, Bruno P. A. Cammue, Karin Thevissen

**Affiliations:** Centre of Microbial and Plant Genetics, KU Leuven, Leuven, Belgium

**Keywords:** antimicrobial peptides, mode of action, antifungal activity, membrane-interaction, review

## Abstract

The incidence of invasive fungal infections is increasing worldwide, resulting in more than 1.6 million deaths every year. Due to growing antifungal drug resistance and the limited number of currently used antimycotics, there is a clear need for novel antifungal strategies. In this context, great potential is attributed to antimicrobial peptides (AMPs) that are part of the innate immune system of organisms. These peptides are known for their broad-spectrum activity that can be directed toward bacteria, fungi, viruses, and/or even cancer cells. Some AMPs act via rapid physical disruption of microbial cell membranes at high concentrations causing cell leakage and cell death. However, more complex mechanisms are also observed, such as interaction with specific lipids, production of reactive oxygen species, programmed cell death, and autophagy. This review summarizes the structure and mode of action of antifungal AMPs, thereby focusing on their interaction with fungal membranes.

## Introduction

In the past two decades, fungal infections have caused severe die-offs and extinctions in wild species, as they comprise the biggest threat for both plant (64%) and animal (72%) species in terms of infection-related species extinctions (Fisher et al., 2012). Moreover, fungal infections are also jeopardizing food security, as they are causing both yield and post-harvest losses (Fisher et al., 2012; Gauthier and Keller, 2013).

In human health, invasive fungal infections (IFIs) are yearly causing 1.6 million deaths worldwide (Fisher et al., 2020). This number is still increasing (i) due to a rise in the number of immunocompromised patients, e.g., patients receiving immunosuppressive therapies in context of cancer treatment or transplantation (Kim, 2016; Bongomin et al., 2017), and (ii) due to an increased use of modern medical devices, such as catheters and implants (Kojic and Darouiche, 2004; Vandecandelaere and Coenye, 2015). Notably, the number of IFI deaths is most likely an underestimation due to poor epidemiological data and misdiagnosis (Bongomin et al., 2017; Spitzer et al., 2017; Benedict et al., 2019). Currently, only three main classes of antifungal drugs are available, namely azoles, polyenes and echinocandins, while antifungal drug resistance is increasing (Fisher et al., 2018; Revie et al., 2018). *Candida* spp. and *Aspergillus* spp. are mainly responsible for IFI with mortality rates up to 50% (Brown et al., 2012; Bongomin et al., 2017). Moreover, the recently identified pathogen *Candida auris* is often multidrug resistant, with some strains resistant to all three available antifungal classes (Lockhart et al., 2017). In addition, both the Centers for Disease Control and Prevention and the World Health Organization recently placed fungal pathogens on their concern list (Centers for Disease Control and Prevention [CDC], 2019; World Health Organization [WHO], 2020). Hence, there is a clear need for novel antifungal strategies.

Antimicrobial peptides (AMPs) are host defense peptides of the nonspecific innate immune system that are part of the first line of immune defense (Hancock and Patrzykat, 2002; Teixeira et al., 2012; Shah et al., 2016, 2019; Contreras et al., 2019). Due to their broad-spectrum activity, selective targeting, multiple modes of action and often limited toxicity to human cells, great potential is attributed to AMPs. AMPs were first described in 1939 by Dubos, who isolated gramicidin from *Bacillus brevis* (Dubos, 1939). Later in the 1980s, cecropins, defensins and magainins were isolated from moths, human neutrophils and amphibians, respectively (Steiner et al., 1981; Ganz et al., 1985; Zasloff, 1987). Currently, over 3000 AMPs are described in the Antimicrobial Peptide Database (Wang et al., 2016), nevertheless only a limited number of AMPs have been studied regarding their mechanism of action. AMPs are present in bacteria, fungi, plants, invertebrates and vertebrates (van der Weerden et al., 2013; Wang et al., 2016; Kumar et al., 2018), and are known for their broad-spectrum that can include activity against bacteria, fungi, viruses, protozoa and/or even cancer cells (Wachinger et al., 1998; do Nascimento et al., 2015; El-Mounadi et al., 2016; Cools et al., 2017a; Parvy et al., 2019; Struyfs et al., 2020). Note that individual AMPs may not act against all these organisms. Apart from their direct action, AMPs can also function indirectly via immunomodulation in higher organisms. Firstly, AMPs can affect the innate immune system, e.g., enhanced wound healing by the salivary peptides histatin 1 and 2 (Oudhoff et al., 2008). Secondly, AMPs can modulate and bridge the adaptive immune response elements, e.g., human β-defensins 1, 2, and 3 (hBD-1,-2, and -3) induce the upregulation of specific cytokines, such as IL-8 and MCP-1, whereas only hBD-1,-2 induce IL-6 and IL-10 (Boniotto et al., 2006). In animals, AMPs are mostly produced by epithelial cells of skin, airways and gastrointestinal tract (Zasloff, 2002), while in plants they preferentially accumulate in the peripheral cell layer (Broekaert et al., 1997; Lay and Anderson, 2005; van der Weerden et al., 2013; Parisi et al., 2019b). AMPs can be constitutively expressed e.g., hBD-1 in epithelial cells (O’Neil et al., 1999); termicin and spinigerin in termites (Lamberty et al., 2001b) and the plant defensin Psd1 in the epidermal tissues and vascular bundles of pea pods (Almeida et al., 2000, 2002). Additionally, AMPs can be expressed upon induction e.g., hBD-2,-3, and -4 in epithelial cells upon infection and inflammation (García et al., 2001; Liu et al., 2003; Sørensen et al., 2003; Gácser et al., 2014); CRAMP in mouse skin upon *Candida albicans* infection (López-García et al., 2005) and plant peptides e.g., AtPDF1.2, AtPDF2.3 in leaves upon microbial invasion (Penninckx et al., 1996; Broekaert et al., 1997; Manners et al., 1998; Thomma and Broekaert, 1998; Lay and Anderson, 2005; van der Weerden et al., 2013; Parisi et al., 2019b).

The focus of this review will be on the structure and mode of action of membrane-interacting antifungal AMPs and on a potential link between their mode of action and membrane contact sites (MCSs), thereby focusing on AMPs of which the mode of action is already described in more detail. Additional information on AMPs can be found in Faruck et al. (2016); Parisi et al. (2019b), Buda De Cesare et al. (2020), and Mookherjee et al. (2020). Gaining insight in their mechanism of action allows to exploit this information for novel therapeutics.

## Structure

Antimicrobial peptides are typically composed of 12 to 54 amino acids (AA) with an overall net positive charge at physiological pH (Teixeira et al., 2012; Mookherjee et al., 2020). However, also negatively charged peptides exist, such as dermcidin (*Homo sapiens*) and maximin H5 (*Bombina maxima*) (Schittek et al., 2001; Lai et al., 2002). In general, up to 50% of the residues are hydrophobic, thereby contributing to an amphipathic conformation that enhances their interaction with the target membrane (Gomes et al., 2018). AMPs are known for their sequence diversity and wide range of secondary structures. Based on their conformation four main groups can be distinguished, i.e., (i) α-helical peptides, (ii) β-sheet peptides with disulfide bridges, (iii) cyclic peptides, and (iv) residue-rich peptides. Firstly, α-helical peptides are usually unstructured in aqueous solvent, whereas in the presence of membranes, they fold into amphiphilic α-helices in which polar and non-polar AA are segregated along the helical axis (Almeida and Pokorny, 2012; Hollmann et al., 2018). The size of α-helical peptides ranges between 12 and 37 AA; they typically contain a kink or central hinge region (Tossi et al., 2000). Examples of α-helical peptides are cathelicidins [e.g., LL-37 (human) and CRAMP (mouse)], cecropins (moths), magainins (amphibians), and melittin (honeybee) (Zasloff, 1987; Gallo et al., 1997; Silvestro et al., 2000; Andrä et al., 2001; Den Hertog et al., 2005; Park and Lee, 2010; Ordonez et al., 2014). Also α-helical peptides with a disulfide bridge have been occasionally described. Their N-terminal region consists of an amphipathic α-helix, whereas the C-terminus contains a disulfide bridge (Almeida and Pokorny, 2012), such as brevinin 2 (*Rana pirica*) and gaegurin 1 (*Rana rugosa*). Secondly, β-sheet peptides with disulfide bridges are characterized by a well-defined number of β-strands that are stabilized by disulfide bonds (Hollmann et al., 2018). These peptides have no or only a few helical domains. Cysteine-containing β-sheet peptides represent a highly diverse group of peptides, mainly represented by defensins. These peptides have been found in fungi, plants, insects and vertebrates (van der Weerden et al., 2013). Defensins have a conserved motif, namely the γ-core, which is an essential structural element that is composed of two antiparallel β-sheets (Brogden, 2005; Sathoff et al., 2019). Although early work described the isolated γ-core as an active antifungal peptide, this is not reproducible for all peptides, as synthetic γ-core peptides have been tested that were not active (Sagaram et al., 2011; Sathoff et al., 2019). For plant defensins, the γ-core comprises surface loop 5 which is essential for antifungal activity (Parisi et al., 2019b). β-sheet peptides can contain two (e.g., tachyplesins and protegrins), three (e.g., mammalian defensins and insect defensins) or four disulfide bridges (e.g., drosomycin and plant defensins) (Broekaert et al., 1997; Parisi et al., 2019b). *Petunia hybrida* defensins 1 and 2 (PhD1,2) are, so far, the only known plant defensins containing five disulfide bridges (Janssen et al., 2003). The presence of disulfide bridges in defensins assures their stability under extreme conditions, such as exposure to high temperatures or in serum, thereby, retaining their biological activity (Osborn et al., 1995; Tavares et al., 2008). Thirdly, cyclic peptides are characterized by a cyclization of the peptide backbone, such as gramicidin S (*Bacillus brevis*), daptomycin (*Streptomyces roseosporus*) and cyclotides, such as kalata B1 and cycloviolacins (Tam et al., 1999; Brogden, 2005; Daly et al., 2009; Almeida and Pokorny, 2012; Slazak et al., 2018). Cyclotides contain three cysteine bridges and a structural motif known as the cyclic cysteine knot, resulting in exceptional stability. Fourthly, residue-rich peptides are characterized by the predominant presence of a particular amino acid, which imposes particular constraints to their structure, such as indolicidin (tryptophan-rich), histatin (histidine-rich) and arasin 1 (proline-rich) (Helmerhorst et al., 1999; Brogden, 2005; Almeida and Pokorny, 2012; Paulsen et al., 2013).

## Mode of Antifungal Action of AMPs

Previously, it was believed that the mode of action of AMPs solely consisted of rapid physical disruption of microbial membranes causing cell death. However, numerous antifungal AMPs have a more complex mechanism of action in which they interact with the surface of fungal cells and affect intracellular targets, leading to e.g., endogenous reactive oxygen species (ROS) production, mitochondrial and vacuolar dysfunction, programmed cell death, autophagy, and cell cycle impairment as described in more detail and summarized in Supplementary Table 1.

### Interaction of AMPs With the Fungal Cell Surface

In the past, the mechanism of action of AMPs was believed to be nonspecific, resulting in pore formation by barrel-stave, carpet or toroidal pore mechanisms. In these mechanisms, (i) peptides form a bundle with a central lumen in the membrane, (ii) accumulate on the bilayer surface, or (iii) insert into the membrane and induce the lipids to bend through the pore resulting in a central lumen consisting of both peptides and lipid head groups (Brogden, 2005; Lazzaro et al., 2020). For certain AMPs, this is indeed the case. For example, using synthetic lipid vesicles, the insect peptide cecropin A (*Hyalophora cecropia*) was demonstrated to form ion channels at low concentrations and pores at high concentrations (Silvestro et al., 2000). It remains, however, unclear whether pore formation of cecropin A is responsible for its antifungal activity. In bacteria, the vertebrate peptide magainin 2 (*Xenopus laevis*) forms pores by the toroidal pore mechanism, whereas in mammalian cells, it forms pores via the carpet mechanism (Imura et al., 2008). Also here, the mode of action on fungal cells remains unclear. Note that membrane disrupting AMPs should not be called pore formers unless there is biophysical evidence that a pore has indeed been formed, as confirmed by e.g., ion conductance and the atomic size of the pore. Nonetheless, there is currently little evidence of antifungal AMPs that form pores *in vivo* or in artificial membranes that closely resemble biological membranes. Nonspecific interaction of antifungal AMPs with antimicrobial membranes is thus not the sole mechanism of action (as discussed in Wilmes et al., 2011). Thevissen et al. (1997, 2000b) were the first to report the existence of specific, high-affinity binding sites for the plant defensins HsAFP1 and DmAMP1 on *Neurospora crassa* hyphae, and demonstrated that binding to these sites is required for their antifungal activity.

#### Interaction of AMPs With the Fungal Cell Membrane

**FIGURE 1:**
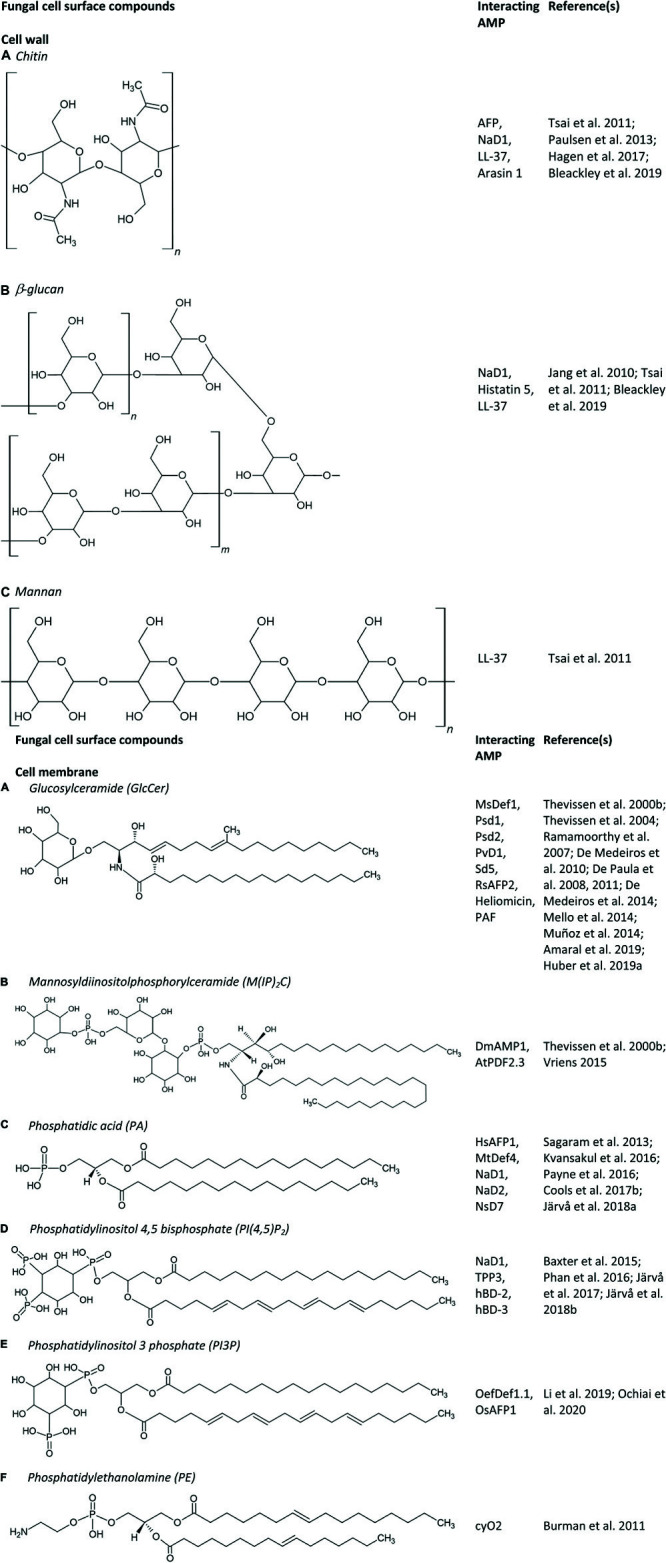
**(A–F)** Fungal cell surface compounds known to play an important role in the antifungal mode of action of AMPs.

**FIGURE 2:**
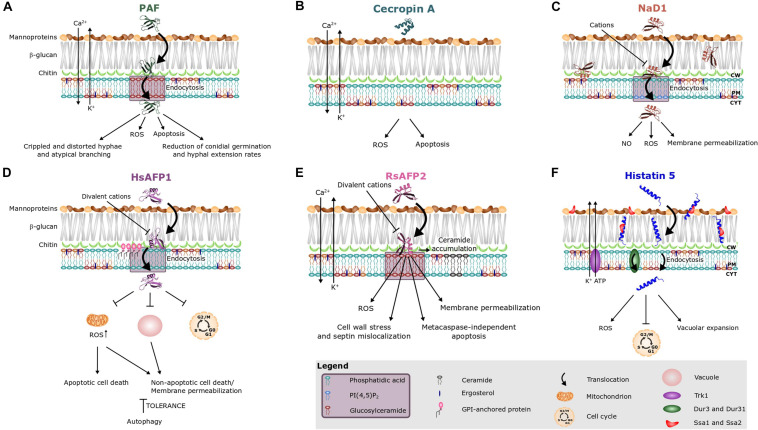
Antifungal mode of action of **(A)** the *Penicillium chrysogenum* antifungal protein PAF and various peptides, i.e., **(B)** cecropin A, **(C)** NaD1, **(D)** HsAFP1, **(E)** RsAFP2, and **(F)** histatin 5. Different peptides are known to interact with different sphingo- and phospholipids, as indicated on the images and in the purple box in the legend. Note that treatment of fungal cells with rather high peptide concentrations can result in aspecific membrane permeabilization (Thevissen et al., 1997, 1999). This phenomenon is not taken into account in this figure.

The first step in the antifungal mechanism of action is thus the interaction of AMPs with their fungal target, which can be the fungal cell membrane and/or the fungal cell wall (see section “Interaction of AMPs With the Fungal Cell Wall”). The former mainly consists of sterols, phospholipids and sphingolipids. Phospholipids are composed of a glycerol backbone coupled to two fatty acids and one polar head group. Numerous phospholipids can be distinguished based on their polar head group, such as phosphatidic acid (PA), phosphatidylcholine, phosphatidylethanolamine (PE), phosphatidylglycerol, phosphatidylserine (PS), and phosphoinositides. Note that binding affinities between AMPs and lipids are rarely measured, and can thus not be compared. Examples of AMPs that interact with PA are the plant defensins HsAFP1, MtDef4, NaD1, NaD2, and NsD7 (Figures 1, 2C,D); some of which interact also with other phospholipids like phosphatidylinositol phosphates (PIPs) and phosphatidylinositol bisphosphates (PIP_2_s) (Figure 1). The RGFRRR-motif of MtDef4 was found to be of importance for the interaction with PA, as mutants devoid of this motif are characterized by reduced binding (Sagaram et al., 2013). Similarly, this motif is present within the sequence of NaD2 and AtPDF2.3 (Cools et al., 2017a). NaD2 is demonstrated to interact with PA as well as with PIPs, as evaluated using lipid overlays and lipid vesicles (Bleackley et al., 2016; Payne et al., 2016). Although lipid overlays can be useful for initial assessment of a potential peptide – lipid interaction, this technique is often unreliable due to the high lipid concentration and the lipid orientation on the strip that are not representative of *in vivo* membranes (Narayan and Lemmon, 2006; Payne et al., 2016). Therefore, potential interactions observed using lipid overlays must be further validated using different techniques, such as ELISA assays, lipid vesicles, structure analysis or deletion mutants of genes that are of importance for the biosynthesis of membrane lipids. It is important to note that by using specific deletion mutants for validating a peptide-membrane component interaction, one cannot distinguish between the membrane component being the genuine peptide interaction partner or only a secondary effector in modulating peptide susceptibility. Therefore, we have listed the specific validation methods that were used in this section. For AtPDF2.3 the interaction with PA has hitherto not been validated. Nevertheless, the presence of an RGFRRR-motif seems not essential for the interaction of AMPs with PA. Recently, using lipid overlays, reverse ELISA assays and lipid vesicles, Cools et al. (2017b) demonstrated the interaction of HsAFP1 with PA, in which histidine at position 32 and arginine at position 52 are important features. Additionally, HsAFP1 also binds, to a lesser extent, to various phosphatidylinositol moieties. AMPs are also able to bind different types of phospholipids to form oligomeric fibrils with different topologies. Note that these fibrils have been produced *in vitro* and there is currently little evidence that they are also produced *in vivo*. In this respect, the plant defensin NsD7 forms a coiled defensin–PA double helix upon interaction with PA, in which the critical residues that enable assembly of the dimers into an oligomer are lysine at position 36 and arginine at position 39 (Kvansakul et al., 2016). NaD1 also interacts with PA, as demonstrated using lipid overlays, lipid vesicles and structure analysis. In contrast, NaD1 and PA form a near-flat, carpet-like oligomeric complex *in vitro* during the initial stages of membrane encounter. Also here, arginine at position 39 is critical for PA binding, oligomerization and fungal cell killing (Järvå et al., 2018a). Additionally, NaD1 also targets PI(4,5)P_2_ (Figure 1), thereby seven dimers of NaD1 bind anionic headgroups of 14 PIP_2_ molecules through a cationic grip configuration. Lysine at position 36 and arginine at position 40 are of importance for the NaD1 – PIP_2_ interaction (Poon et al., 2014; Bleackley et al., 2016). This interaction seems to be essential for NaD1’s activity on tumor cells, whereas it does not seem to be essential for its antifungal activity. Nevertheless, it can be challenging to identify relevant interaction partners, since AMPs, in particular plant defensins, have multiple targets so eliminating one can weaken but does not necessarily abolish antifungal activity. Likewise, TPP3 adopts a cationic grip dimer conformation that mediates binding of PIP_2_ *in vitro* (Baxter et al., 2015). Also NsD7 interacts with PI(4,5)P_2_. In this case the oligomeric topology formed by NsD7- PIP_2_ is nearly identical to the previously determined NaD1–PIP_2_ complex (Järvå et al., 2017). Both hBD-2 and hBD-3 are also able to interact with PI(4,5)P_2_ as evaluated using lipid overlays, liposome pulldown assays and structure determination in the case of hBD-2, and using lipid overlays and lipid vesicles in case of hBD-3 (Phan et al., 2016; Järvå et al., 2018b). HBD-2 dimers specifically interact with two distinct PIP_2_-binding sites to permeabilize fungal cell membranes (Järvå et al., 2018b). Note that, PI(4,5)P_2_ is localized in the inner leaflet of the cell membrane (Borges-Araújo and Fernandes, 2020), therefore, AMPs will first target other cell wall/cell membrane components. In contrast, glucose-containing glucosylceramide (GlcCer) is also found at the fungal cell wall (see section “Interaction of AMPs With the Fungal Cell Wall”) (Rodrigues et al., 2000). The olive tree defensin OefDef1.1 and the rice defensin OsAFP1 are known to interact with both PI3P and PI5P, and PI3P, respectively, as validated using lipid overlays (Figure 1; Li et al., 2019; Ochiai et al., 2020). The cycloviolacin O2 (cyO2) is known to selectively target and disrupt membranes containing PE, as evaluated using lipid vesicles (Figure 1; Burman et al., 2011).

Apart from AMPs that interact with phospholipids, several AMPs have been identified that interact with sphingolipids. Sphingolipids, together with sterols, typically form membrane rafts, so-called lipid rafts, that are enriched in specific domains in fungal plasma membranes (Hurst and Fratti, 2020). Sphingolipids consist of a ceramide backbone linked to a polar head group. Based on this polar head group, they are classified in two groups, being phosphosphingolipids and glycosphingolipids. The phosphosphingolipid mannosyldiinositolphosphorylceramide [M(IP)_2_C] is the major sphingolipid in *Saccharomyces cerevisiae* (Dickson et al., 1997), and plays an important role in the antifungal mechanism of action of DmAMP1 and AtPDF2.3 (documented via an ELISA assay and/or specific yeast deletion mutants) (Figure 1; Thevissen et al., 2000a; Vriens, 2015). GlcCer is the most common glycosphingolipid in fungi, except for *S. cerevisiae* and *Candida glabrata*, since they do not produce GlcCer (Saito et al., 2006; Tavares et al., 2008). So far, GlcCer was identified to be of importance in the antifungal mode of action of numerous AMPs, such as the plant defensins MsDef1, Psd1, Psd2, PvD1, Sd5, and RsAFP2, the insect defensin-like peptide heliomicin, the *Aspergillus giganteus* peptide AFP and the *Penicillium chrysogenum* antifungal protein PAF (Figures 1, 2A,E; Thevissen et al., 2004; De Paula et al., 2008, 2011; De Medeiros et al., 2014; Mello et al., 2014; Muñoz et al., 2014; Amaral et al., 2019; Huber et al., 2019). The involvement of GlcCer in the mode of action of these AMPs was established via lipid vesicles (e.g., Psd1, Psd2, Sd5, RsAFP2, and PAF), ELISA assays (RsAFP2 and heliomicin) and deletion mutants (e.g., AFP, MsDef1, Psd1, PvD1, RsAFP2, heliomicin, and PAF) (Thevissen et al., 2000a, 2004; Ramamoorthy et al., 2007; De Paula et al., 2008, 2011; De Medeiros et al., 2010, 2014; Mello et al., 2014; Muñoz et al., 2014; Vriens, 2015; Amaral et al., 2019; Huber et al., 2019). Often multiple biochemical tests are used to confirm the relevance of these potential interactions. Note that RsAFP2 interacts with GlcCers isolated from *Pichia pastoris* but not with those of soybean nor human GlcCers, whereas heliomicin interacts with GlcCers isolated from both *P. pastoris* and soybean but not with human GlcCers. Moreover, RsAFP2 and heliomicin were suggested to interact with different motifs of fungal GlcCer, as the interaction of RsAFP2 with fungal GlcCer could not be competed for by heliomicin and vice versa (Thevissen et al., 2004). Interestingly, although most sphingolipids are non-essential for cell survival, they are key regulators of pathogenicity in a variety of fungi, such as *C. albicans*, which makes them essential for *in vivo* infections (Noble et al., 2010). Therefore, the incidence of *in vivo* resistance development against sphingolipid-targeting AMPs is expected to be minimal as resistant mutants with defective sphingolipid biosynthesis are not virulent (Del Poeta et al., 2014).

Antimicrobial proteins can also interact with plasma membrane proteins. Human lactoferrin targets the plasma membrane Pma1p H^+^-ATPase, thereby perturbating cation homeostasis (cfr. supra) (Andrés et al., 2016, 2019).

#### Interaction of AMPs With the Fungal Cell Wall

Apart from the interaction of AMPs with the fungal cell membrane, AMPs can also interact with the fungal cell wall which is mainly composed of glucans, chitin and glycosylated proteins (Garcia-Rubio et al., 2020). Note that there are differences in cell wall composition between fungal species, e.g., galactomannan makes up a major part of the *Aspergillus* cell wall. Consequently, its presence is currently used as a diagnostic test for invasive aspergillosis (Mercier et al., 2018). GlcCer is also found at the fungal cell wall (Rodrigues et al., 2000), explaining the interaction of RsAFP2 with the fungal cell wall (Thevissen et al., 2012). Moreover, AMPs can also interact with other cell wall specific compounds. Recently, the interaction of both chitin and β-glucan with the tobacco plant defensin NaD1 was described (Figures 1, 2C). Herein, the yeast cell wall protects target cells against the antifungal activity of NaD1, as the killing of spheroplasts occurred at lower concentrations as compared to cells with intact walls (Bleackley et al., 2019). Note that AMPs can have multiple fungal targets, as NaD1 also interacts with PA and PIP_2_ (Figure 2C) (cfr. infra). The salivary peptide histatin 5 interacts with β-glucan, as well as with the heat shock proteins Ssa1p and Ssa2p that are present in the cell wall and cell membrane of *C. albicans* cells (Figures 1, 2F; Li et al., 2003; Jang et al., 2010). Binding with the latter is required for the fungicidal activity of histatin 5 (Li et al., 2003). Similarly, the human β-defensins hBD-2 and hBD-3 bind Ssa1p and Ssa2p as part of their antifungal mode of action (Vylkova et al., 2006). The human neutrophil defensin 1 (HNP-1) competes with histatin 5 for Ssa1p and Ssa2p binding sites, however, using deletion mutants, it could be demonstrated that HNP-1 does not require Ssa1p or Ssa2p for its fungicidal activity (Edgerton et al., 2000; Vylkova et al., 2006). The human cathelicidin LL-37 preferentially interacts with mannan, but also with chitin or glucan (Figure 1; Tsai et al., 2011). Likewise, the proline-rich antimicrobial peptide arasin 1 from spider crab also binds chitin (Figure 1). However, this chitin-binding property may also arise from its involvement in host wound healing (Paulsen et al., 2013). Also AFP binds chitin as validated *in vitro* (Figure 1; Hagen et al., 2007).

#### Influence of Cations on the Initial Association of AMPs With the Fungal Cell Surface

The initial association of cationic AMPs with anionic bilayers is mostly directed by electrostatic interactions due to their net charge being opposed to that of the target membrane (von Deuster and Knecht, 2011; Alvares et al., 2017). Therefore, the presence of cations can significantly reduce the antifungal activity of AMPs, as demonstrated in case of e.g., AFP, coprisin (dung beetle), DmAMP1, HNP-1, HsAFP1, indolicidin (cattle), NaD1, OefDef1.1, P113 (a 12-amino-acid fragment of histatin 5), RsAFP2, and hBD-1,-2 (Figures 2C–E; Osborn et al., 1995; Goldman et al., 1997; Bals et al., 1998; Edgerton et al., 2000; Lee et al., 2003, 2012, 2014; Theis et al., 2003; Bleackley et al., 2019; Li et al., 2019; Cheng et al., 2020). Similarly, in yeast mutants lacking Agp2p, a plasma membrane regulator of polyamine and carnitine transport, the antifungal activity of NaD1 is significantly reduced. Deletion of AGP2 was suggested to result in an accumulation of cationic molecules at the cell surface, thereby repelling cationic AMPs (Bleackley et al., 2014). Since the hydrolysis of the cell wall by zymolyase treatment restored the activity of NaD1 in high salt conditions (100 mM NaCl), the loss of antifungal activity at elevated salt concentrations was recently attributed to the sequestration of cations by fungal cell wall polysaccharides (Bleackley et al., 2019). Note that Bleackley et al. (2019) only evaluated the effect of monovalent cations, being Na^+^, thereby reporting the plant defensin DmAMP1 to be salt tolerant. In contrast, Osborn et al. (1995) earlier reported that the antifungal activity of DmAMP1 against both *N. crassa* and *Fusarium culmorum* is significantly reduced in the presence of both 1 and 5 mM Ca^2+^ or Mg^2+^. Hence, it remains unclear if divalent cations are also sequestrated by fungal cell wall polysaccharides. In case of the plant defensin HsAFP1, the presence of divalent cations specifically reduced the uptake of the peptide, thereby inhibiting its antifungal activity (Figure 2D; Struyfs et al., 2020). The salt-sensitivity of most peptides (at least *in vitro*) is probably one of the main factors why only few AMPs have been further developed into a commercial antifungal or antibacterial product. However, there are few reports on the efficacy of AMPs upon systemic administration, thus acting in biological cation-containing fluids like blood, in preclinical models (see conclusion section of this review). For example, the plant defensin RsAFP2 demonstrated *in vivo* efficacy in a prophylactic murine model of candidiasis upon intravenous administration (Tavares et al., 2008). Only a limited number of AMPs are salt tolerant of which most originate from marine organisms (Lee et al., 1997; Fedders et al., 2008). Also the antifungal activity of dermcidin, expressed in sweat glands, is maintained at high salt concentrations that resemble human sweat conditions. Since dermcidin is negatively charged at physiological pH, electrostatic interactions with membrane lipids might not be of importance for its antifungal activity (Schittek et al., 2001). Exceptionally, the presence of divalent cations can also enhance the antifungal activity of AMPs. Histatin 5 binds multiple cations, including Zn^2+^ which induces dimerization of the peptide. Zn^2+^ that is abundantly present in saliva, significantly increases killing activity of histatin 5 in both *C. albicans* and *C. glabrata* (Norris et al., 2020).

### Downstream Pathways Involved in the Antifungal Mode of Action of AMPs

#### Potential AMP Uptake

Previously, it was believed that the mode of action of AMPs solely consisted of simple pore formation that resulted in rapid killing. However, antifungal AMPs can have a more complex mechanism of action that includes signaling pathways and intracellular targets. Hitherto, the mechanism of action of only a limited number of AMPs has been described. Amongst them are AMPs that (i) are internalized upon their initial interaction with the fungal cell, without causing fungal membrane disruption (e.g., histatin 5, HsAFP1, MtDef4, NaD1, *Neosartorya fischeri* antifungal protein NFAP, OefDef1.1, Psd1 and PAF) (Figures 2A,D,F) or (ii) remain outside the cell (e.g., RsAFP2) (Figure 2E; Oberparleiter et al., 2003; Kumar et al., 2011; Thevissen et al., 2012; El-Mounadi et al., 2016; Cools et al., 2017b; Hajdu et al., 2019; Li et al., 2019). Note that internalization is not essential for inducing intracellular mechanisms, as RsAFP2 is not internalized, but does induce e.g., production of ROS and programmed cell death (Aerts et al., 2007; Thevissen et al., 2012). The internalization process of AMPs can be species-specific. In *Fusarium oxysporum* and *Fusarium virguliforme*, the translocation of OefDef1.1 to the cytoplasm occurs in both germlings and conidia. However, in *Botrytis cinerea*, uptake only occurs in germlings (Li et al., 2019). In *N. crassa*, the internalization of MtDef4 depends on the presence of phospholipase D, whereas this is not the case in *Fusarium graminearum* (Sagaram et al., 2011; El-Mounadi et al., 2016). The uptake of AMPs is often an active, energy requiring process, pointing to endocytosis as in case of HsAFP1, histatin 5, MtDef4, NaD1, NFAP, and PAF (Figures 2A,C,D,F; Oberparleiter et al., 2003; Jang et al., 2010; Kumar et al., 2011; Puri and Edgerton, 2014; El-Mounadi et al., 2016; Cools et al., 2017b; Hayes et al., 2018; Hajdu et al., 2019). Indeed, in *N. crassa*, internalization of MtDef4 is energy dependent and requires endocytosis, while in *F. graminearum*, uptake is only partially energy dependent (El-Mounadi et al., 2016). Internalization of histatin 5 can occur via three different pathways (Figure 2F) being (i) endocytosis, (ii) transportation into the fungal cell via the polyamine transporters Dur3p and Dur31p in an energy-dependent process, and (iii) membrane lytic effects if the histatin concentration, estimated 15–30 μM in saliva, exceeds 30 μM (Kumar et al., 2011; Puri and Edgerton, 2014). Deletion of the GPI-anchor remodeling enzyme *BST1* results in increased resistance of *S. cerevisiae* cells to HsAFP1 treatment due to a compromised HsAFP1 internalization pathway, pointing to an important role for GPI-anchor remodeling enzymes in HsAFP1’s internalization (Figure 2D; Struyfs et al., 2020).

#### Production of Endogenous ROS and the Induction of Programmed Cell Death

Similar to certain antimycotics (e.g., amphotericin B), numerous AMPs, such as the plant defensin ApDef1, cecropin A, coprisin, histatin 5, HsAFP1, human lactoferrin, the plant defensin LpDef1, NaD1, OefDef1.1, the honeybee venom peptide melittin, PAF, the *Penicillium chrysogenum* antifungal protein C (PAFC), PvD1, RsAFP2 and the centipede peptide scolopendin induce the production of ROS (Figure 2; Leiter et al., 2005; Andrés et al., 2008; Park and Lee, 2010; Aerts et al., 2011; Mello et al., 2011; Lee et al., 2012, 2016; Galgóczy et al., 2013; Choi et al., 2014; Yun and Lee, 2016; Soares et al., 2017; Vriens et al., 2017; Holzknecht et al., 2020). Excessive cellular levels of ROS can cause oxidative damage to proteins, nucleic acids, lipids, membranes and organelles, which in turn can lead to activation of cell death processes such as programmed cell death (Redza-Dutordoir and Averill-Bates, 2016). Programmed cell death is a tightly regulated and highly conserved process of cell death in which plasma membrane integrity is initially preserved and intracellular content is degraded in a programmed manner. Numerous AMPs are known to induce programmed cell death, such as ApDef1, cecropin A, coprisin, hBD-2, HsAFP1, human lactoferrin, melittin, NPAF, OsAFP1, PAF, RsAFP2, and scolopendin (Figures 2A,B,D,E; Leiter et al., 2005; Andrés et al., 2008; Yount et al., 2009; Park and Lee, 2010; Aerts et al., 2011; Lee et al., 2012, 2016; Thevissen et al., 2012; Galgóczy et al., 2013; Yun and Lee, 2016; Soares et al., 2017; Ochiai et al., 2018). In *C. albicans*, RsAFP2 treatment results in cell wall stress, septin mislocalization and increased ceramide levels, the latter having been previously demonstrated to result in programmed cell death (Thevissen et al., 2012; Rego et al., 2014). Indeed, RsAFP2 induces programmed cell death in a metacaspase-independent (Mca1) manner (Aerts et al., 2007). Nevertheless, the exact mechanism of programmed cell death induction is hitherto unclear. In contrast, ApDef1 induces chromatin condensation, resulting in cell death via metacaspase-dependent programmed cell death (Soares et al., 2017). Similarly, metacaspase-dependent programmed cell death is induced upon treatment with coprisin, preceded by the exposure of PS on the outer leaflet of the plasma membrane, dysfunctional mitochondrial transmembrane potential (Δψm) and cytochrome C release (Lee et al., 2012). Human lactoferrin induces metacaspase-dependent programmed cell death in *C. albicans*, as lactoferrin-treated cells exhibited PS exposure, chromatin condensation and DNA degradation (Andrés et al., 2008; Acosta-Zaldívar et al., 2016). Also scolopendin treatment induces programmed cell death via metacaspase activation, as evidenced by PS externalization, cytochrome C release, de-energization, chromatin condensation and DNA fragmentation (Lee et al., 2016). Cecropin A triggers programmed cell death characterized by PS externalization, cytochrome C release, dissipation of Δψm and DNA fragmentation (Yun and Lee, 2016). Upon treatment with hBD-2, apoptotic cell death follows PS accessibility in *C. albicans* (Yount et al., 2009). Likewise, melittin induces programmed cell death in *C. albicans*, as evidenced by PS externalization, and DNA and nuclear fragmentation (Park and Lee, 2010). Note that, spinigerin induces programmed cell death in a caspase independent manner in *Leishmania donovan*. So far, it remains unclear if this also occurs in fungal cells (Sardar et al., 2013).

#### Mitochondrial Dysfunction

Moreover, AMP treatment can also result in mitochondrial dysfunction, as demonstrated for coprisin, ETD151 (analog of the antifungal insect defensin heliomicin), HsAFP1, human lactoferrin, LpDef1 and scolopendin (Figure 2D; Aerts et al., 2011; Lee et al., 2012, 2016; Puri and Edgerton, 2014; Vieira et al., 2015; Acosta-Zaldívar et al., 2016; Aumer et al., 2020). ETD151 does not interact directly with complexes I and III of the mitochondrial respiratory chain. Therefore, it was suggested that mitochondrial dysfunction results from mitochondrial membrane perturbation (Aumer et al., 2020). Co-incubation of HsAFP1 and sodium azide, which blocks the respiratory electron transport chain at complex IV, antagonized the antifungal activity of HsAFP1. This points to the indispensability of a functional respiratory chain for HsAFP1 antifungal action (Aerts et al., 2011). Likewise, treatment of *C. albicans* cells with sodium azide also results in a decreased susceptibility to coprisin, hBD-2, hBD-3, and histatin 5 (Helmerhorst et al., 1999; Vylkova et al., 2007; Lee et al., 2012). *S. cerevisiae rho*^0^ deletion mutants, lacking the mitochondrial genome, are more resistant to NaD1. Hence, mitochondrial respiratory activity is required for the antifungal activity of NaD1 (Hayes et al., 2013).

#### ATP Efflux

Another feature of the mechanism of action of AMPs is the release of ATP from target cells, as in the case of histatin 5, LL-37, hBD-2, and hBD-3 (Figure 2F; Koshlukova et al., 1999; Den Hertog et al., 2005; Vylkova et al., 2007). In *C. albicans*, histatin 5 causes a drastic reduction of intracellular ATP content, which results from the efflux of ATP (Koshlukova et al., 1999). Similarly, killing by both hBD-2 and hBD-3 involves ATP release. The magnitude and profile of ATP efflux are most similar between histatin 5 and hBD-2, as opposed to hBD-3 in which the maximal ATP release at 60 min peptide treatment only reached 50% of the ATP efflux level caused by histatin 5 (Vylkova et al., 2007). Notably, no gross membrane disruption occurs upon ATP efflux when tested with these AMPs, except in case of LL-37.

#### Cell Cycle Impairment

Antimicrobial peptides can also affect cell cycle progression, which is the case for ApDef1, histatin 5, HsAFP1 and Psd1 (Figures 2D,F; Baev et al., 2002; Lobo et al., 2007; Soares et al., 2017; Struyfs et al., 2020). Upon HsAFP1 treatment of *S. cerevisiae* cells, cell cycle impairment was observed. Nonetheless, this impairment was also present upon treatment with the inactive HsAFP1 mutant, HsAFP1[H32A][R52A] (Cools et al., 2017b), when tested at equimolar concentrations. Hence, these findings indicate that HsAFP1-induced cell cycle impairment is not linked to its antifungal activity (Struyfs et al., 2020). Therefore, cell cycle impairment was considered as a non-specific secondary effect rather than the primary cause of HsAFP1’s killing activity.

#### Disruption of Cation Homeostasis

Furthermore, AMPs can also abolish cation homeostasis in target cells, herein mostly intracellular levels of Ca^2+^ and K^+^ ions are disturbed. Ca^2+^ plays an important role in signal transduction pathways, where it acts as a second messenger e.g., in programmed cell death, and is mainly stored in the endoplasmic reticulum (ER) as well as in mitochondria (Kinjo and Schnetkamp, 2005). In contrast, K^+^ is the major cytoplasmic cation, thereby mainly controlling the ionic strength of the cytoplasm, and its depletion is critical for progress on the apoptotic pathway (Baev et al., 2004). DmAMP1, RsAFP2, PAF, and cecropin A induce both K^+^ efflux and Ca^2+^ influx upon interaction with fungal target cells (Figures 2A,B,E; Thevissen et al., 1996, 1999; Kaiserer et al., 2003; Binder et al., 2010, 2015; Yun and Lee, 2016), whereas scolopendin, MsDef1 and MtDef4 induce Ca^2+^ influx and human lactoferrin and histatin 5 induce K^+^ efflux (Figure 2F; Baev et al., 2004; Andrés et al., 2008; Muñoz et al., 2014; Lee et al., 2017). Interestingly, the potassium transporter Trk1 is required for the antifungal mode of action of histatin 5, as it was suggested to provide the essential pathway for ATP loss (Baev et al., 2004).

#### Induction of Autophagy and Vacuolar Dysfunction

Recently, AMPs were also found to induce autophagy, which is a cytoprotective process in which intracellular material is digested in the vacuole (Carmona-Gutierrez et al., 2018). Notably, excessive levels of autophagy have been linked with cell death (Liu and Levine, 2015). Moderate HsAFP1 doses induce autophagy in *S. cerevisiae* cells, probably as a fungal tolerance/pro-survival mechanism against the HsAFP1 action (Figure 2D; Struyfs et al., 2020). Since functional vacuoles are indispensable for the induction of autophagy (Martínez-Muñoz and Kane, 2008; Sampaio-Marques et al., 2019), they might play a role in governing tolerance to HsAFP1. Indeed, at high HsAFP1 doses vacuoles are affected, as demonstrated by the increased vacuolar pH, possibly resulting in impaired autophagy, and therefore in effective killing of *S. cerevisiae* cells. After NbD6 treatment the vacuoles of both *S. cerevisiae* and *F. graminearum* cells fused into one large vacuole, while the peptide remained on the cell surface. Though such large vacuole resembles those that form in an autophagic response, a possible link between both must be validated (Parisi et al., 2019a). In contrast, after SBI6 treatment, cells contained multiple fragmented vacuoles, pointing to vacuolar disruption (Parisi et al., 2019a). While functional vacuoles reportedly seem to govern tolerance to HsAFP1, deletions in vacuolar genes result in increased resistance to NbD6 and SBI6 (Aerts et al., 2011; Parisi et al., 2019a). These findings indicate that these peptides do not impair vacuolar function, but that their antifungal effect rather depends on a properly functioning vacuole (Parisi et al., 2019a).

#### Potential Link Between the Antifungal Mode of Action of AMPs and MCSs

In eukaryotic cells, incompatible biochemical processes are spatially separated by intracellular compartmentalization in organelles. Most organelles are physically connected via MCSs (Kohler et al., 2020). Notably, at MCSs, organelle membranes are closely apposed and tethered, however, not fused (Scorrano et al., 2019). MCSs play a critical role in inter-organelle communication that is essential in maintaining cellular homeostasis, including lipid synthesis and trafficking, and intracellular signaling (e.g., Ca^2+^) (Scorrano et al., 2019). Although there is currently no evidence of MCS involvement in the mode of action of AMPs, it could be linked with their antifungal activity. In particular, MCSs play an important role in the regulation of autophagy, as the MCSs between the phagophore and the ER allow the direct transfer of lipids that are required for membrane extension, and thus the progression of autophagy (Molino et al., 2017; Kohler et al., 2020). Moreover, the endosomal protein Vps13p transiently localizes to nucleus-vacuole junctions in yeast (Lang et al., 2015). Interestingly, the *S. cerevisiae* Δ*vps13* deletion mutant is 4-fold more resistant to HsAFP1 (Aerts et al., 2011), pointing to a potential link between MCSs and HsAFP1 functioning. Furthermore, MCSs are involved in Ca^2+^ homeostasis at the ER-mitochondria interface (Phillips and Voeltz, 2016), and disruption of cation homeostasis is part of the antifungal activity of numerous AMPs. Hence, MCSs could be involved in the antifungal mode of action of AMPs. This hypothetical link should be further investigated by different techniques, such as super-resolution microscopy, transmission electron microscopy, FRET-based reporters or proximity ligation assays, in the presence and absence of AMPs to evaluate their potential effect on MCSs (Nascimbeni et al., 2017; Scorrano et al., 2019).

## Conclusion

Antimicrobial peptides are part of the innate immune system of numerous organisms and are known for their broad-spectrum activity. Their structure is highly diverse and grouped according to their conserved 3D structure. In the past, the mode of action of AMPs was generally considered as nonspecific, resulting in rapid physical disruption of microbial cell membranes causing cell leakage and death. However, AMPs can also exert a more complex mechanism of action. Many AMPs specifically interact with the fungal cell wall and/or cell membrane, with various sphingolipids and phospholipids identified as AMP targets. Upon target interaction, peptides can be internalized or can remain at the outside of the fungal cell. Regardless of potential uptake, antifungal peptides can affect intracellular targets, thereby resulting in a multitude of actions such as ROS production, programmed cell death, mitochondrial dysfunction, disruption of cation homeostasis, ATP efflux, cell cycle impairment, autophagy, and vacuolar dysfunction. Moreover, MCSs might be linked with the mode of action of antifungal AMPs.

Elucidating the mode of action of AMPs is a crucial step to exploit their application potential. Hitherto, the activity of certain AMPs is demonstrated *in vivo* as exemplified by RsAFP2’s activity in a prophylactic murine model of candidiasis and by the histatin 5 derivative P113’s activity against oral candidiasis that completed clinical phase IIb (Tavares et al., 2008; Brunetti et al., 2016; Håkansson et al., 2019; Mookherjee et al., 2020). Inactivation of AMPs by cations upon systemic administration could be limiting for commercial applications, however, alternative administration routes, such as topical administration, in which the inactivation of AMPs by cations is of less importance, could be of interest. For example, ropocamptide, developed by ProMore Pharma, Sweden, is based on LL-37 [known to be inactivated by 100 mM NaCl (Turner et al., 1998)] and has recently passed clinical phase IIb study in patients with venous leg ulcers to improve chronic would healing (Thapa et al., 2020; Promore Pharma, 2021). As compared to small molecules, peptides mostly have a greater efficacy, selectivity and specificity (Hummel et al., 2006; McGregor, 2008, Henninot et al., 2018). Nonetheless, they are also more prone to degradation as compared to small molecule-based drugs. To overcome this limitation, non-natural amino acids and/or cyclization can be introduced to enhance peptide stability (Ding et al., 2020). Numerous AMP-based drugs are currently evaluated in clinical trials [as reviewed in Mookherjee et al. (2020)] for a variety of potential applications, such as antimicrobial therapies and immunomodulatory therapies. Apart from directly exploiting antifungal peptides and their derivatives, insight in the mechanism of action of AMPs can also be used to facilitate the efficient design and development of novel AMP-like antifungal compounds with high selectivity. This can be exemplified by fungal GlcCer-specific camelid single domain antibodies, which were designed to mimic RsAFP2’s interaction with GlcCer and hence, are active against a broad spectrum of plant pathogenic fungi (De Coninck et al., 2017). This points to the potential of AMPs in a myriad of novel applications.

## Author Contributions

KT coordinated the review of the manuscript. BC revised the review of the manuscript. CS contributed to writing of the manuscript, redaction, and revisions. All authors contributed to the article and approved the submitted version.

## Conflict of Interest

The authors declare that the research was conducted in the absence of any commercial or financial relationships that could be construed as a potential conflict of interest.

## References

[B1] Acosta-Zaldívar M. Andrés M. T. Rego A. Pereira C. S. Fierro J. F. Côrte-Real M. (2016). Human lactoferrin triggers a mitochondrial- and caspase-dependent regulated cell death in *Saccharomyces cerevisiae*. *Apoptosis* 21 163–173. 10.1007/s10495-015-1199-9 26577769

[B2] Aerts A. M. Bammens L. Govaert G. Carmona-Gutierrez D. Madeo F. Cammue B. P. A. (2011). The antifungal plant defensin HsAFP1 from *Heuchera sanguinea* induces apoptosis in *Candida albicans*. *Front. Microbiol.* 2:47. 10.3389/fmicb.2011.00047 21993350 PMC3128936

[B3] Aerts A. M. François I. E. J. A. Bammens L. Cammue B. P. A. Smets B. Winderickx J. (2006). Level of M(IP)_2_C sphingolipid affects plant defensin sensitivity, oxidative stress resistance and chronological life-span in yeast. *FEBS Lett.* 580 1903–1907. 10.1016/j.febslet.2006.02.061 16527275

[B4] Aerts A. M. François I. E. J. A. Meert E. M. K. Li Q. T. Cammue B. P. A. Thevissen K. (2007). The antifungal activity of RsAFP2, a plant defensin from *Raphanus sativus*, involves the induction of reactive oxygen species in *Candida albicans*. *J. Mol. Microbiol. Biotechnol.* 13 243–247. 10.1159/000104753 17827975

[B5] Allen A. Snyder A. K. Preuss M. Nielsen E. E. Shah D. M. Smith T. J. (2008). Plant defensins and virally encoded fungal toxin KP4 inhibit plant root growth. *Planta* 227 331–339. 10.1007/s00425-007-0620-1 17849147

[B6] Almeida M. S. Cabral K. M. S. Kurtenbach E. Almeida F. C. L. Valente A. P. (2002). Solution structure of *Pisum sativum* defensin 1 by high resolution NMR: plant defensins, identical backbone with different mechanisms of action. *J. Mol. Biol.* 315 749–757. 10.1006/jmbi.2001.5252 11812144

[B7] Almeida M. S. Cabral K. M. S. Zingali R. B. Kurtenbach E. (2000). Characterization of two novel defense peptides from pea (*Pisum sativum*) seeds. *Arch. Biochem. Biophys.* 378 278–286. 10.1006/abbi.2000.1824 10860545

[B8] Almeida P. F. Pokorny A. (2012). Interactions of antimicrobial peptides with lipid bilayers. *Biophys. J.* 5 189–222. 10.1016/B978-0-12-374920-8.00515-4

[B9] Alvares D. S. Viegas T. G. Neto J. R. (2017). Lipid-packing perturbation of model membranes by pH-responsive antimicrobial peptides. *Biophys. Rev*. 9 669–682. 10.1007/s12551-017-0296-0 28853007 PMC5662038

[B10] Amaral V. S. G. Fernandes C. M. Felício M. R. Valle A. S. Quintana P. G. Almeida C. C. (2019). Psd2 pea defensin shows a preference for mimetic membrane rafts enriched with glucosylceramide and ergosterol. *Biochim. Biophys. Acta Biomembr.* 1861 713–728. 10.1016/j.bbamem.2018.12.020 30639288

[B11] Andrä J. Berninghausen O. Leippe M. (2001). Cecropins, antibacterial peptides from insects and mammals, are potently fungicidal against *Candida albicans*. *Med. Microbiol. Immunol.* 189 169–173. 10.1007/s430-001-8025-x 11388616

[B12] Andrés M. T. Acosta-Zaldívar M. Fierro J. F. (2016). Antifungal mechanism of action of lactoferrin: identification of H^+^-ATPase (P3A-type) as a new apoptotic-cell membrane receptor. *Antimicrob. Agents Chemother.* 60 4206–4216. 10.1128/AAC.03130-15 27139463 PMC4914641

[B13] Andrés M. T. Acosta-Zaldívar M. González-Seisdedos J. Fierro J. F. (2019). Cytosolic acidification is the first transduction signal of lactoferrin-induced regulated cell death pathway. *Int. J. Mol. Sci.* 20 5838–5854. 10.3390/ijms20235838 31757076 PMC6928705

[B14] Andrés M. T. Viejo-Díaz M. Fierro J. F. (2008). Human lactoferrin induces apoptosis-like cell death in *Candida albicans*: critical role of K^+^-channel-mediated K^+^ efflux. *Antimicrob. Agents Chemother.* 52 4081–4088. 10.1128/AAC.01597-07 18710913 PMC2573133

[B15] Aumer T. Voisin B. N. Knobloch T. Landon C. Bulet P. (2020). Impact of an antifungal insect defensin on the proteome of the phytopathogenic fungus *Botrytis cinerea*. *J. Proteome. Res.* 19 1131–1146. 10.1021/acs.jproteome.9b00638 31967833

[B16] Baev D. Li X. S. Dong J. Keng P. Edgerton M. (2002). Human salivary histatin 5 causes disordered volume regulation and cell cycle arrest in *Candida albicans*. *Infect. Immun.* 70 4777–4784. 10.1128/IAI.70.9.4777-4784.2002 12183519 PMC128240

[B17] Baev D. Rivetta A. Vylkova S. Sun J. N. Zeng G. F. Slayman C. L. (2004). The TRK1 potassium transporter is the critical effector for killing of *Candida albicans* by the cationic protein, histatin 5. *J. Biol. Chem.* 279 55060–55072. 10.1074/jbc.M411031200 15485849

[B18] Bals R. Wang X. Wu Z. Freeman T. Bafna V. Zasloff M. (1998). Human β-defensin 2 is a salt-sensitive peptide antibiotic expressed in human lung. *J. Clin. Investig.* 102 874–880. 10.1172/JCI2410 9727055 PMC508952

[B19] Baxter A. A. Richter V. Lay F. T. Poon I. K. H. Adda C. G. Veneer P. K. (2015). The tomato defensin TPP3 binds phosphatidylinositol (4,5)-bisphosphate via a conserved dimeric cationic grip conformation to mediate cell lysis. *Mol. Cell. Biol.* 35 1964–1978. 10.1128/mcb.00282-15 25802281 PMC4420927

[B20] Benedict K. Jackson B. R. Chiller T. Beer K. D. (2019). Estimation of direct healthcare costs of fungal diseases in the United States. *Clin. Infect. Dis.* 68 1791–1797. 10.1093/cid/ciy776 30204844 PMC6409199

[B21] Benincasa M. Scocchi M. Pacor S. Tossi A. Nobili D. Basaglia G. (2006). Fungicidal activity of five cathelicidin peptides against clinically isolated yeasts. *J. Antimicr. Chemother.* 58 950–959. 10.1093/jac/dkl382 17023499

[B22] Binder U. Benèina M. Fizil A. Batta G. Chhillar A. K. Marx F. (2015). Protein kinase A signaling and calcium ions are major players in PAF mediated toxicity against *Aspergillus niger*. *FEBS Lett.* 589 1266–1271. 10.1016/j.febslet.2015.03.037 25882631 PMC4424949

[B23] Binder U. Chu M. Read N. D. Marx F. (2010). The antifungal activity of the *Penicillium chrysogenum* protein PAF disrupts calcium homeostasis in *Neurospora crassa*. *Eukaryot. Cell* 9 1374–1382. 10.1128/EC.00050-10 20622001 PMC2937333

[B24] Bleackley M. R. Dawson C. S. McKenna J. A. Quimbar P. Hayes B. M. E. van der Weerden N. L. (2017). Synergistic activity between two antifungal proteins, the plant defensin NaD1 and the bovine pancreatic trypsin inhibitor. *mSphere* 2:e00390-17. 10.1128/mSphere.00390-17 29062897 PMC5646242

[B25] Bleackley M. R. Dawson C. S. Payne J. A. E. Harvey P. J. Rosengren K. J. Quimbar P. (2019). The interaction with fungal cell wall polysaccharides determines the salt tolerance of antifungal plant defensins. *Cell Surface* 5:100026. 10.1016/j.tcsw.2019.100026 32743142 PMC7389181

[B26] Bleackley M. R. Payne J. A. E. Hayes B. M. E. Durek T. Craik D. J. Shafee T. M. A. (2016). *Nicotiana alata* defensin chimeras reveal differences in the mechanism of fungal and tumor cell killing and an enhanced antifungal variant. *Antimicrob. Agents Chemother.* 60 6302–6312. 10.1128/AAC.01479-16 27503651 PMC5038239

[B27] Bleackley M. R. Wiltshire J. L. Perrine-Walker F. Vasa S. Burns R. L. van der Weerden N. L. (2014). Agp2p, the plasma membrane transregulator of polyamine uptake, regulates the antifungal activities of the plant defensin NaD1 and other cationic peptides. *Antimicrob. Agents Chemother.* 58 2688–2698. 10.1128/AAC.02087-13 24566173 PMC3993230

[B28] Bongomin F. Gago S. Oladele R. O. Denning D. W. (2017). Global and multi-national prevalence of fungal diseases—estimate precision. *J. Fungi* 3 1–29. 10.3390/jof3040057 29371573 PMC5753159

[B29] Boniotto M. Jordan W. J. Eskdale J. Tossi A. Antcheva N. Crovella S. (2006). Human β-defensin 2 induces a vigorous cytokine response in peripheral blood mononuclear cells. *Antimicrob. Agents Chemother.* 50 1433–1441. 10.1128/AAC.50.4.1433-1441.2006 16569862 PMC1426918

[B30] Borges-Araújo L. Fernandes F. (2020). Structure and lateral organization of phosphatidylinositol 4,5-bisphosphate. *Molecules* 25:3885. 10.3390/molecules25173885 32858905 PMC7503891

[B31] Broekaert W. F. Cammue B. P. A. De Bolle M. F. C. Thevissen K. De Samblanx G. W. Osborn R. W. (1997). Antimicrobial peptides from plants. *Crit. Rev. Plant Sci.* 16 297–323. 10.1080/07352689709701952

[B32] Brogden K. A. (2005). Antimicrobial peptides: pore formers or metabolic inhibitors in bacteria? *Nat. Rev. Microbiol*. 3 238–250. 10.1038/nrmicro1098 15703760

[B33] Brown G. D. Denning D. W. Gow N. A. R. Levitz S. M. Netea M. G. White T. C. (2012). Hidden killers: human fungal infections. *Sci. Transl. Med.* 4:165rv13. 10.1126/scitranslmed.3004404 23253612

[B34] Brunetti J. Falciani C. Roscia G. Pollini S. Bindi S. Scali S. (2016). *In vitro* and *in vivo* efficacy, toxicity, bio-distribution and resistance selection of a novel antibacterial drug candidate. *Sci. Rep.* 6:26077. 10.1038/srep26077 27169671 PMC4864329

[B35] Buda De Cesare G. Cristy S. A. Garsin D. A. Lorenz M. C. (2020). Antimicrobial peptides: a new frontier in antifungal therapy. *mBio* 11:e2123-20. 10.1128/mBio.02123-20 33144376 PMC7642678

[B36] Burman R. Strömstedt A. A. Malmsten M. Göransson U. (2011). Cyclotide-membrane interactions: defining factors of membrane binding, depletion and disruption. *Biochim. Biophys. Acta* 1808 2665–2673. 10.1016/j.bbamem.2011.07.004 21787745

[B37] Carmona-Gutierrez D. Bauer M. A. Zimmermann A. Aguilera A. Austriaco N. Ayscough K. (2018). Guidelines and recommendations on yeast cell death nomenclature. *Microb. Cell* 5 4–31. 10.15698/mic2018.01.607 29354647 PMC5772036

[B38] Centers for Disease Control and Prevention [CDC] (2019). *U.S. Department of Health and Human Services. Drug-Resistant Candida auris.* Available online at: https://www.cdc.gov/drugresistance/pdf/threats-report/candida-auris-508.pdf (accessed on February 2021)

[B39] Cheng K. T. Wu C. L. Yip B. S. Chih Y. H. Peng K. L. Hsu S. Y. (2020). The interactions between the antimicrobial peptide P-113 and living *Candida albicans* cells shed light on mechanisms of antifungal activity and resistance. *Int. J. Mol. Sci.* 21:2654. 10.3390/ijms21072654 32290246 PMC7178208

[B40] Cho Y. Turner J. S. Dinh N. N. Lehrer R. I. (1998). Activity of protegrins against yeast-phase *Candida albicans*. *Infect. Immun*. 66 2486–2493. 10.1128/IAI.66.6.2486-2493.1998 9596706 PMC108228

[B41] Choi H. Hwang J. S. Lee D. G. (2014). Identification of a novel antimicrobial peptide, scolopendin 1, derived from centipede *Scolopendra subspinipes mutilans* and its antifungal mechanism. *Insect Mol. Biol.* 23 788–799. 10.1111/imb.12124 25209888

[B42] Cohen L. Moran Y. Sharon A. Segal D. Gordon D. Gurevitz M. (2009). Drosomycin, an innate immunity peptide of *Drosophila melanogaster*, interacts with the fly voltage-gated sodium channel. *J. Biol. Chem.* 284 23558–23563. 10.1074/jbc.M109.023358 19574227 PMC2749130

[B43] Contreras G. Shirdel I. Braun M. S. Wink M. (2019). Defensins: transcriptional regulation and function beyond antimicrobial activity. *Dev. Comp. Immunol.* 104 103556. 10.1016/j.dci.2019.103556 31747541

[B44] Cools T. L. Struyfs C. Cammue B. P. A. Thevissen K. (2017a). Antifungal plant defensins: increased insight in their mode of action as a basis for their use to combat fungal infections. *Future Microbiol.* 12 441–454. 10.2217/fmb-2016-0181 28339295

[B45] Cools T. L. Struyfs C. Drijfhout J. W. Kucharíková S. Lobo Romero C. Van Dijck P. (2017b). A linear 19-mer plant defensin-derived peptide acts synergistically with caspofungin against *Candida albicans* biofilms. *Front. Microbiol.* 8:2051. 10.3389/fmicb.2017.02051 29104569 PMC5655031

[B46] Cools T. L. Vriens K. Struyfs C. Verbandt S. Ramada M. H. S. Brand G. D. (2017c). The antifungal plant defensin HsAFP1 Is a phosphatidic acid-interacting peptide inducing membrane permeabilization. *Front. Microbiol.* 8:2295. 10.3389/fmicb.2017.02295 29209301 PMC5702387

[B47] Czajlik A. Holzknecht J. Galgóczy L. Tóth L. Poór P. Ördög A. (2021). Solution structure, dynamics, and new antifungal aspects of the cysteine-rich miniprotein PAFC. *Int. J. Mol. Sci.* 22:1183. 10.3390/ijms22031183 33504082 PMC7865535

[B48] Daly N. L. Rosengren J. K. Craik D. J. (2009). Discovery, structure and biological activities of cyclotides. *Adv. Drug Deliv. Rev.* 61 918–930. 10.1016/j.addr.2009.05.003 19470399

[B49] De Coninck B. Verheesen P. Vos C. M. Van Daele I. De Bolle M. F. Vieira J. V. (2017). Fungal glucosylceramide-specific camelid single domain antibodies are characterized by broad spectrum antifungal activity. *Front. Microbiol.* 8:1059. 10.3389/fmicb.2017.01059 28659884 PMC5469901

[B50] De Medeiros L. N. Angeli R. Sarzedas C. G. Barreto-Bergter E. Valente A. P. Kurtenbach E. (2010). Backbone dynamics of the antifungal Psd1 pea defensin and its correlation with membrane interaction by NMR spectroscopy. *Biochim. Biophys. Acta Biomembr.* 1798 105–113. 10.1016/j.bbamem.2009.07.013 19632194

[B51] De Medeiros L. N. Domitrovic T. De Andrade P. C. Faria J. Bergter E. B. Weissmuller G. (2014). Psd1 binding affinity toward fungal membrane components as assessed by SPR: the role of glucosylceramide in fungal recognition and entry. *Biopolymers* 102 456–464. 10.1002/bip.22570 25283273

[B52] De Paula V. S. Razzera G. Barreto-Bergter E. Almeida F. C. L. Valente A. P. (2011). Portrayal of complex dynamic properties of sugarcane defensin 5 by NMR: multiple motions associated with membrane interaction. *Structure* 19 26–36. 10.1016/j.str.2010.11.011 21220113

[B53] De Paula V. S. Razzera G. Medeiros L. Miyamoto C. A. Almeida M. S. Kurtenbach E. (2008). Evolutionary relationship between defensins in the *Poaceae* family strengthened by the characterization of new sugarcane defensins. *Plant Mol. Biol.* 68 321–335. 10.1007/s11103-008-9372-y 18618271

[B54] Del Poeta M. Nimrichter L. Rodrigues M. L. Luberto C. (2014). Synthesis and biological properties of fungal glucosylceramide. *PLoS Pathog.* 10:e1003832. 10.1371/journal.ppat.1003832 24415933 PMC3887071

[B55] Den Hertog A. L. Van Marle J. Van Veen H. A. Van’t Hof W. Bolscher J. G. M. Veerman E. C. I. (2005). Candidacidal effects of two antimicrobial peptides: histatin 5 causes small membrane defects, but LL-37 causes massive disruption of the cell membrane. *Biochem. J.* 388 689–695. 10.1042/BJ20042099 15707390 PMC1138977

[B56] Dickson R. C. Nagiec E. E. Wells G. B. Nagiec M. M. Lester R. L. (1997). Synthesis of mannose-(inositol-P)_2_ -ceramide, the major sphingolipid in *Saccharomyces cerevisiae*, requires the *IPT1* (*YDR072c*) gene. *J. Biol. Chem.* 272 29620–29625. 10.1074/jbc.272.47.29620 9368028

[B57] Ding Y. Ting J. P. Liu J. Al-Azzam S. Pandya P. Afshar S. (2020). Impact of non-proteinogenic amino acids in the discovery and development of peptide therapeutics. *Amino Acids* 52 1207–1226. 10.1007/s00726-020-02890-9 32945974 PMC7544725

[B58] Do N. Weindl G. Grohmann L. Salwiczek M. Koksch B. Korting H. C. (2014). Cationic membrane-active peptides - anticancer and antifungal activity as well as penetration into human skin. *Exp. Dermatol.* 23 326–331. 10.1111/exd.12384 24661024

[B59] do Nascimento V. V. Mello ÉO. Carvalho L. P. De Melo E. J. T. Carvalho A. O. Fernandes K. V. S. (2015). PvD1 defensin, a plant antimicrobial peptide with inhibitory activity against *Leishmania amazonensis*. *Biosci. Rep.* 35:e00248. 10.1042/BSR20150060 26285803 PMC4613715

[B60] Dong J. Vylkova S. Li X. S. Edgerton M. (2003). Calcium blocks fungicidal activity of human salivary histatin 5 through disruption of binding with *Candida albicans*. *J. Dent. Res.* 82 748–752. 10.1177/154405910308200917 12939362

[B61] Dracatos P. M. Payne J. Di Pietro A. Anderson M. A. Plummer K. M. (2016). Plant defensins NaD1 and NaD2 induce different stress response pathways in fungi. *Int. J. Mol. Sci*. 17:1473. 10.3390/ijms17091473 27598152 PMC5037751

[B62] Dubos R. J. (1939). Studies on a bactericidal agent extracted from a soil *Bacillus*. *J. Exp. Med.* 70 11–17. 10.1084/jem.70.1.1 19870886 PMC2133780

[B63] Edgerton M. Koshlukova S. E. Araujo M. W. Patel R. C. Dong J. Bruenn J. A. (2000). Salivary histatin 5 and human neutrophil defensin 1 kill *Candida albicans* via shared pathways. *Antimicrob. Agents Chemother.* 44 3310–3306. 10.1128/aac.44.12.3310-3316.2000 11083633 PMC90198

[B64] El-Mounadi K. Islam K. T. Hernández-Ortiz P. Read N. D. Shah D. M. (2016). Antifungal mechanisms of a plant defensin MtDef4 are not conserved between the ascomycete fungi *Neurospora crassa* and *Fusarium graminearum*. *Mol. Microbiol.* 100 542–559. 10.1111/mmi.13333 26801962

[B65] Faruck M. O. Yusof F. Chowdhury S. (2016). An overview of antifungal peptides derived from insect. *Peptides* 80 80–88. 10.1016/j.peptides.2015.06.001 26093218

[B66] Fedders H. Michalek M. Grötzinger J. Leippe M. (2008). An exceptional salt-tolerant antimicrobial peptide derived from a novel gene family of haemocytes of the marine invertebrate *Ciona intestinalis*. *Biochem. J.* 416 65–75. 10.1042/BJ20080398 18598239

[B67] Fehlbaum P. Bulet P. Michaut L. Lagueux M. Broekaert W. F. Hetru C. (1994). Insect immunity: septic injury of *Drosophila* induces the synthesis of a potent antifungal peptide with sequence homology to plant antifungal peptides. *J. Biol. Chem.* 269 33159–33163. 10.1016/S0021-9258(20)30111-3 7806546

[B68] Feng Z. Jiang B. Chandra J. Ghannoum M. Nelson S. Weinberg A. (2005). Human beta-defensins: differential activity against Candidal species and regulation by *Candida albicans*. *J. Dent. Res.* 84 445–450. 10.1177/154405910508400509 15840781

[B69] Ferket K. K. A. Levery S. B. Park C. Cammue B. P. A. Thevissen K. (2003). Isolation and characterization of *Neurospora crassa* mutants resistant to antifungal plant defensins. *Fungal Genet. Biol.* 40 176–185. 10.1016/S1087-1845(03)00085-9 14516770

[B70] Fisher M. Henk D. Briggs C. Brownstein J. S. Madoff L. C. McCraw S. L. (2012). Emerging fungal threats to animal, plant and ecosystem health. *Nature* 484 186–194. 10.1038/nature10947 22498624 PMC3821985

[B71] Fisher M. C. Gurr S. J. Cuomo C. A. Blehert D. S. Jin H. Stukenbrock E. H. (2020). Threats posed by the fungal kingdom to humans, wildlife, and agriculture. *mBio* 11:e00449-20. 10.1128/mBio.00449-20 32371596 PMC7403777

[B72] Fisher M. C. Hawkins N. J. Sanglard D. Gurr S. J. (2018). Worldwide emergence of resistance to antifungal drugs challenges human health and food security. *Science* 360 739–742. 10.1126/science.aap7999 29773744

[B73] Gácser A. Tiszlavicz Z. Németh T. Seprényi G. Mándi Y. (2014). Induction of human defensins by intestinal Caco-2 cells after interactions with opportunistic *Candida* species. *Microbes Infect.* 16 80–85. 10.1016/j.micinf.2013.09.003 24095867

[B74] Galgóczy L. Kovács L. Karácsony Z. Virágh M. Hamari Z. Vágvölgyi C. (2013). Investigation of the antimicrobial effect of *Neosartorya fischeri* antifungal protein (NFAP) after heterologous expression in *Aspergillus nidulans*. *Microbiolgy* 159 411–419. 10.1099/mic.0.061119-0 23197172

[B75] Gallo R. L. Kim K. J. Bernfield M. Kozak C. A. Zanetti M. Merluzzi L. (1997). Identification of CRAMP, a cathelin-related antimicrobial peptide expressed in the embryonic and adult mouse. *J. Biol. Chem.* 272 13088–13093. 10.1074/jbc.272.20.13088 9148921

[B76] Gank K. D. Yeaman M. R. Kojima S. Yount N. Y. Park H. Edwards J. E. (2008). SSD1 is integral to host defense peptide resistance in *Candida albicans*. *Eukaryot. Cell* 7 1318–1327. 10.1128/EC.00402-07 18515753 PMC2519774

[B77] Ganz T. Selsted M. E. Szklarek D. Harwig S. S. Daher K. Bainton D. F. (1985). Defensins. Natural peptide antibiotics of human neutrophils. *J. Clin. Investig.* 76 1427–1435. 10.1172/JCI112120 2997278 PMC424093

[B78] Gao B. Zhu S. Y. (2008). Differential potency of drosomycin to *Neurospora crassa* and its mutant: implications for evolutionary relationship between defensins from insects and plants. *Insect Mol. Biol.* 17 405–411. 10.1111/j.1365-2583.2008.00810.x 18651922

[B79] García J. R. C. Krause A. Schulz S. Rodríguez-Jiménez F. J. Klüver E. Adermann K. (2001). Human β-defensin 4: a novel inducible peptide with a specific salt-sensitive spectrum of antimicrobial activity. *FASEB J.* 15 1819–1821. 10.1096/fj.00-0865fje 11481241

[B80] Garcia-Rubio R. de Oliveira H. C. Rivera J. Trevijano-Contador N. (2020). The fungal cell wall: *Candida*, *Cryptococcus*, and *Aspergillus* species. *Front. Microbiol*. 10:2993. 10.3389/fmicb.2019.02993 31993032 PMC6962315

[B81] Gauthier G. M. Keller N. P. (2013). Crossover fungal pathogens: the biology and pathogenesis of fungi capable of crossing kingdoms to infect plants and humans. *Fungal Genet. Biol.* 61 146–157. 10.1016/j.fgb.2013.08.016 24021881

[B82] Goldman M. J. Anderson M. G. Stolzenberg E. D. Kari P. U. Zasloff M. Wilson J. M. (1997). Human β-defensin-1 is a salt-sensitive antibiotic in lung that is inactivated in cystic fibrosis. *Cell* 88 553–560. 10.1016/S0092-8674(00)81895-4 9038346

[B83] Gomes B. Augusto M. T. Felício M. R. Hollmann A. Franco O. L. Gonçalves S. (2018). Designing improved active peptides for therapeutic approaches against infectious diseases. *Biotechnol. Adv.* 36 415–429. 10.1016/j.biotechadv.2018.01.004 29330093

[B84] Gonçalves S. Silva P. M. Felício M. R. de Medeiros L. N. Kurtenbach E. Santos N. C. (2017). Psd1 effects on *Candida albicans* planktonic cells and biofilms. *Front. Cell. Infect. Microbiol.* 7:249. 10.3389/fcimb.2017.00249 28649561 PMC5465278

[B85] Hagen S. Marx F. Ram A. F. Meyer V. (2007). The antifungal protein AFP from *Aspergillus giganteus* inhibits chitin synthesis in sensitive fungi. *Appl. Environ. Microbiol.* 73 2128–2134. 10.1128/AEM.02497-06 17277210 PMC1855660

[B86] Hajdu D. Huber A. Czajlik A. Tóth L. Kele Z. Kocsubé S. (2019). Solution structure and novel insights into phylogeny and mode of action of the *Neosartorya* (*Aspergillus*) *fischeri* antifungal protein (NFAP). *Int. J. Biol. Macromol.* 129 511–522. 10.1016/j.ijbiomac.2019.02.016 30738898

[B87] Håkansson J. Ringstad L. Umerska A. Johansson J. Andersson T. Boge L. (2019). Characterization of the *in vitro*, *ex vivo*, and *in vivo* efficacy of the antimicrobial peptide DPK-060 used for topical treatment. *Front. Cell. Infect. Microbiol.* 9:174. 10.3389/fcimb.2019.00174 31192163 PMC6548878

[B88] Hancock R. E. W. Patrzykat A. (2002). Clinical development of cationic antimicrobial peptides: from natural to novel antibiotics. *Curr. Drug Targets Infect. Disord.* 2 79–83. 10.2174/1568005024605855 12462155

[B89] Hayes B. M. E. Bleackley M. R. Anderson M. A. van der Weerden N. L. (2018). The plant defensin NaD1 enters the cytoplasm of *Candida albicans* via endocytosis. *J. Fungi* 4:20. 10.3390/jof4010020 29415460 PMC5872323

[B90] Hayes B. M. E. Bleackley M. R. Wiltshire J. L. Anderson M. A. Traven A. van der Weerden N. L. (2013). Identification and mechanism of action of the plant defensin NaD1 as a new member of the antifungal drug arsenal against *Candida albicans*. *Antimicrob. Agents Chemother.* 57 3667–3675. 10.1128/AAC.00365-13 23689717 PMC3719733

[B91] Helmerhorst E. J. Breeuwer P. Van’t Hof W. Walgreen-Weterings E. Oomen L. C. J. M. Veerman E. C. I. (1999). The cellular target of histatin 5 on *Candida albicans* is the energized mitochondrion. *J. Biol. Chem.* 274 7286–7291. 10.1074/jbc.274.11.7286 10066791

[B92] Helmerhorst E. J. Venuleo C. Beri A. Oppenheim F. G. (2005). *Candida glabrata* is unusual with respect to its resistance to cationic antifungal proteins. *Yeast* 22 705–714. 10.1002/yea.1241 16034806

[B93] Henninot A. Collins J. C. Nuss J. M. (2018). The current state of peptide drug discovery: back to the future? *J. Med. Chem.* 61 1382–1414. 10.1021/acs.jmedchem.7b00318 28737935

[B94] Henriques S. T. Huang Y. H. Castanho M. A. R. B. Bagatolli L. A. Sonza S. Tachedjian G. (2012). Phosphatidylethanolamine binding is a conserved feature of cyclotide-membrane interactions. *J. Biol. Chem.* 287 33629–33643. 10.1074/jbc.M112.372011 22854971 PMC3460461

[B95] Hollmann A. Martinez M. Maturana P. Semorile L. C. Maffia P. C. (2018). Antimicrobial peptides: interaction with model and biological membranes and synergism with chemical antibiotics. *Front. Chem*. 6:204. 10.3389/fchem.2018.00204 29922648 PMC5996110

[B96] Holzknecht J. Kühbacher A. Papp C. Farkas A. Váradi G. Marcos J. F. (2020). The *Penicillium chrysogenum* Q176 antimicrobial protein PAFC effectively inhibits the growth of the opportunistic human pathogen *Candida albicans*. *J. Fungi* 6:141. 10.3390/jof6030141 32824977 PMC7557831

[B97] Huber A. Galgóczy L. Váradi G. Holzknecht J. Kakar A. Malanovic N. (2020). Two small, cysteine-rich and cationic antifungal proteins from *Penicillium chrysogenum*: a comparative study of PAF and PAFB. *Biochim. Biophys. Acta Biomembr.* 1862:183246. 10.1016/j.bbamem.2020.183246 32142818 PMC7138148

[B98] Huber A. Hajdu D. Bratschun-Khan D. Gáspári Z. Varbanov M. Philippot S. (2018). New antimicrobial potential and structural properties of PAFB: a cationic, cysteine-rich protein from *Penicillium chrysogenum* Q176. *Sci. Rep*. 8:1751. 10.1038/s41598-018-20002-2 29379111 PMC5788923

[B99] Huber A. Oemer G. Malanovic N. Lohner K. Kovács L. Salvenmoser W. (2019). Membrane sphingolipids regulate the fitness and antifungal protein susceptibility of *Neurospora crassa*. *Front. Microbiol*. 10:605. 10.3389/fmicb.2019.00605 31031714 PMC6471014

[B100] Hummel G. Reineke U. Reimer U. (2006). Translating peptides into small molecules. *Mol. Biosyst*. 2 499–508. 10.1039/b611791k 17216031

[B101] Hurst L. R. Fratti R. A. (2020). Lipid rafts, sphingolipids, and ergosterol in yeast vacuole fusion and maturation. *Front. Cell Dev. Biol*. 8:539. 10.3389/fcell.2020.00539 32719794 PMC7349313

[B102] Ikonomova S. P. Moghaddam-Taaheri P. Wang Y. Doolin M. T. Stroka K. M. Hube B. (2020). Effects of histatin 5 modifications on antifungal activity and kinetics of proteolysis. *Prot. Sci.* 29 480–493. 10.1002/pro.3767 31675138 PMC6954697

[B103] Imura Y. Choda N. Matsuzaki K. (2008). Magainin 2 in action: distinct modes of membrane permeabilization in living bacterial and mammalian cells. *Biophys. J.* 95 5757–5765. 10.1529/biophysj.108.133488 18835901 PMC2599864

[B104] Jang W. S. Bajwa J. S. Sun J. N. Edgerton M. (2010). Salivary histatin 5 internalization by translocation, but not endocytosis, is required for fungicidal activity in *Candida albicans*. *Mol. Microbiol.* 77 354–370. 10.1111/j.1365-2958.2010.07210.x 20487276 PMC2909388

[B105] Janssen B. J. C. Schirra H. J. Lay F. T. Anderson M. A. Craik D. J. (2003). Structure of petunia hybrida defensin 1, a novel plant defensin with five disulfide bonds. *Biochemistry* 42 8214–8222. 10.1021/bi034379o 12846570

[B106] Järvå M. Lay F. T. Hulett M. D. Kvansakul M. (2017). Structure of the defensin NsD7 in complex with PIP2 reveals that defensin : lipid oligomer topologies are dependent on lipid type. *FEBS Lett*. 591 2482–2490. 10.1002/1873-3468.12761 28741756

[B107] Järvå M. Lay F. T. Phan T. K. Humble C. Poon I. K. H. Bleackley M. R. (2018a). X-Ray structure of a carpet-like antimicrobial defensin-phospholipid membrane disruption complex. *Nat. Commun.* 9:1962. 10.1038/s41467-018-04434-y 29773800 PMC5958116

[B108] Järvå M. Phan T. K. Lay F. T. Caria S. Kvansakul M. Hulett M. D. (2018b). Human-defensin 2 kills *Candida albicans* through phosphatidylinositol 4,5-bisphosphate–mediated membrane permeabilization. *Sci. Adv.* 4 979–1004. 10.1126/sciadv.aat0979 30050988 PMC6059731

[B109] Joly S. Maze C. McCray P. B. Guthmiller J. M. (2004). Human β-defensins 2 and 3 demonstrate strain-selective activity against oral microorganisms. *J. Clin. Microbiol.* 42 1024–1029. 10.1128/JCM.42.3.1024-29 15004048 PMC356847

[B110] Jung S. I. Finkel J. S. Solis N. V. Chaili S. Mitchell A. P. Yeaman M. R. (2013). Bcr1 functions downstream of Ssd1 to mediate antimicrobial peptide resistance in *Candida albicans*. *Eukaryot. Cell* 12 411–419. 10.1128/EC.00285-12 23314964 PMC3629773

[B111] Kaiserer L. Oberparleiter C. Weiler-Görz R. Burgstaller W. Leiter E. Marx F. (2003). Characterization of the *Penicillium chrysogenum* antifungal protein PAF. *Arch. Microbiol.* 180 204–210. 10.1007/s00203-003-0578-8 12856109

[B112] Kim J. Y. (2016). Human fungal pathogens: why should we learn? *Korea J. Microbiol.* 54 145–148. 10.1007/s12275-016-0647-8 26920875

[B113] Kinjo T. G. Schnetkamp P. P. M. (2005). “Ca^2+^ chemistry, storage and transport in biologic systems,” in *Voltage-Gated Calcium Channels. Molecular Biology Intelligence Unit*, (Boston, MA: Springer). 10.1007/0-387-27526-6_1

[B114] Kohler V. Aufschnaiter A. Büttner S. (2020). Closing the gap: membrane contact sites in the regulation of autophagy. *Cells* 9:1184. 10.3390/cells9051184 32397538 PMC7290522

[B115] Kojic E. M. Darouiche R. O. (2004). Candida infections of medical devices. *Clin. Microbiol. Rev*. 17 255–267. 10.1128/CMR.17.2.255-267.2004 15084500 PMC387407

[B116] Koshlukova S. E. Lloyd T. L. Araujo M. W. B. Edgerton M. (1999). Salivary histatin 5 induces non-lytic release of ATP from *Candida albicans* leading to cell death. *J. Biol. Chem.* 274 18872–18879. 10.1074/jbc.274.27.18872 10383383

[B117] Kovács L. Virágh M. Takó M. Papp T. Vágvölgyi C. Galgóczy L. (2011). Isolation and characterization of *Neosartorya fischeri* antifungal protein (NFAP). *Peptides* 32 1724–1731. 10.1016/j.peptides.2011.06.022 21741420

[B118] Kovács R. Holzknecht J. Hargitai Z. Papp C. Farkas A. Borics A. (2019). *In vivo* applicability of *Neosartorya fischeri* antifungal protein 2 (NFAP2) in treatment of vulvovaginal candidiasis. *Antimicrob. Agents Chemother*. 63:e01777-18. 10.1128/AAC.01777-18 30478163 PMC6355578

[B119] Kovács R. Nagy F. Tóth Z. Forgács L. Tóth L. Váradi G. (2021). The *Neosartorya fischeri* antifungal protein 2 (NFAP2): a new potential weapon against multidrug-resistant *Candida auris* biofilms. *Int. J. Mol. Sci.* 22:771. 10.3390/ijms22020771 33466640 PMC7828714

[B120] Krishnakumari V. Rangaraj N. Nagaraj R. (2009). Antifungal activities of human beta-defensins hBD-1 to hBD-3 and their C-terminal analogs Phd1 to Phd3. *Antimicrob. Agents Chemother.* 53 256–260. 10.1128/AAC.00470-08 18809937 PMC2612179

[B121] Kumar P. Kizhakkedathu J. N. Straus S. K. (2018). Antimicrobial peptides: diversity, mechanism of action and strategies to improve the activity and biocompatibility *in vivo*. *Biomolecules* 8:4. 10.3390/biom8010004 29351202 PMC5871973

[B122] Kumar R. Chadha S. Saraswat D. Bajwa J. S. Li R. A. Conti H. R. (2011). Histatin 5 uptake by *Candida albicans* utilizes polyamine transporters Dur3 and Dur31 proteins. *J. Biol. Chem.* 286 43748–43758. 10.1074/jbc.M111.311175 22033918 PMC3243549

[B123] Kvansakul M. Lay F. T. Adda C. G. Veneer P. K. Baxter A. A. Phan T. K. (2016). Binding of phosphatidic acid by NsD7 mediates the formation of helical defensin-lipid oligomeric assemblies and membrane permeabilization. *Proc. Nat. Acad. Sci. U.S.A.* 113 11202–11207. 10.1073/pnas.1607855113 27647905 PMC5056070

[B124] Lai R. Liu H. Lee W. H. Zhang Y. (2002). An anionic antimicrobial peptide from toad *Bombina maxima*. *Biochem. Biophys. Res. Commun.* 295 796–799. 10.1016/S0006-291X(02)00762-3 12127963

[B125] Lamberty M. Caille A. Landon C. Tassin-Moindrot S. Hetru C. Bulet P. (2001a). Solution structures of the antifungal heliomicin and a selected variant with both antibacterial and antifungal activities. *Biochemistry* 40 11995–12003. 10.1021/bi0103563 11580275

[B126] Lamberty M. Zachary D. Lanot R. Bordereau C. Robert A. Hoffmann J. A. (2001b). Insect immunity. Constitutive expression of a cysteine-rich antifungal and a linear antibacterial peptide in a termite insect. *J. Biol. Chem.* 276 4085–4092. 10.1074/jbc.M002998200 11053427

[B127] Landon C. Barbault F. Legrain M. Menin L. Guenneugues M. Schott V. (2003). Lead optimization of antifungal peptides with 3D NMR structures analysis. *Protein Sci.* 13 703–713. 10.1110/ps.03404404 14978308 PMC2286723

[B128] Landon C. Sodano P. Hetru C. Hoffmann J. Ptak M. (1997). Solution structure of drosomycin, the first inducible antifungal protein from insects. *Protein Sci.* 6 1878–1884. 10.1002/pro.5560060908 9300487 PMC2143780

[B129] Lang A. B. Peter A. T. J. Walter P. Kornmann B. (2015). ER-mitochondrial junctions can be bypassed by dominant mutations in the endosomal protein Vps13. *J. Cell Biol.* 210 883–890. 10.1083/jcb.201502105 26370498 PMC4576869

[B130] Lay F. T. Anderson M. A. (2005). Defensins – components of the innate immune system in plants. *Curr. Protein Pept. Sci.* 6 85–101. 10.2174/1389203053027575 15638771

[B131] Lay F. T. Mills G. D. Poon I. K. H. Cowieson N. P. Kirby N. K. Baxter A. A. (2012). Dimerization of plant defensin NaD1 enhances its antifungal activity. *J. Biol. Chem.* 287 19961–19972. 10.1074/jbc.M111.331009 22511788 PMC3370180

[B132] Lay F. T. Schirra H. J. Scanlon M. J. Anderson M. A. Craik D. J. (2003). The three-dimensional solution structure of NaD1, a new floral defensin from *Nicotiana alata* and its application to a homology model of the crop defense protein alfAFP. *J. Mol. Biol.* 325 175–188. 10.1016/S0022-2836(02)01103-8 12473460

[B133] Lazzaro B. P. Zasloff M. Rolff J. (2020). Antimicrobial peptides: application informed by evolution. *Science* 368:5480. 10.1126/science.aau5480 32355003 PMC8097767

[B134] Lee D. G. Kim H. K. Kim S. A. Park Y. Park S. C. Jang S. H. (2003). Fungicidal effect of indolicidin and its interaction with phospholipid membranes. *Biochem. Biophys. Res. Commun.* 305 305–310. 10.1016/s0006-291x(03)00755-1 12745074

[B135] Lee H. Hwang J. S. Lee D. G. (2016). Scolopendin 2 leads to cellular stress response in *Candida albicans*. *Apoptosis* 21 856–865. 10.1007/s10495-016-1254-1 27207682

[B136] Lee H. Hwang J. S. Lee D. G. (2017). Scolopendin, an antimicrobial peptide from centipede, attenuates mitochondrial functions and triggers apoptosis in *Candida albicans*. *Biochem. J.* 474 635–645. 10.1042/BCJ20161039 28008133

[B137] Lee I. H. Zhao C. Cho Y. Harwig S. S. L. Cooper E. L. Lehrer R. I. (1997). Clavanins, α-helical antimicrobial peptides from tunicate hemocytes. *FEBS Lett.* 400 158–162. 10.1016/S0014-5793(96)01374-9 9001389

[B138] Lee J. Hwang J. S. Hwang I. S. Cho J. Lee E. Kim Y. (2012). Coprisin-induced antifungal effects in *Candida albicans* correlate with apoptotic mechanisms. *Free Radic. Biol. Med.* 52 2302–2311. 10.1016/j.freeradbiomed.2012.03.012 22542795

[B139] Lee J. Lee D. Choi H. Kim H. H. Kim H. Hwang J. S. (2014). Structure-activity relationships of the intramolecular disulfide bonds in coprisin, a defensin from the dung beetle. *BMB Rep.* 47 625–630. 10.5483/BMBRep.2014.47.11.262 24393527 PMC4281341

[B140] Lehrer R. I. Ganz T. Szklarek D. Selsted M. E. (1988). Modulation of the *in vitro* candidacidal activity of human neutrophil defensins by target cell metabolism and divalent cations. *J. Clin. Invest.* 81 1829–1835. 10.1172/JCI113527 3290255 PMC442632

[B141] Leiter É Szappanos H. Oberparleiter C. Kaiserer L. Csernoch L. Pusztahelyi T. (2005). Antifungal protein PAF severely affects the integrity of the plasma membrane of *Aspergillus nidulans* and induces an apoptosis-like phenotype. *Antimicrob. Agents Chemother.* 49 2445–2453. 10.1128/AAC.49.6.2445-2453.2005 15917545 PMC1140496

[B142] Li H. Velivelli S. L. S. Shah D. M. (2019). Antifungal potency and modes of action of a novel olive tree defensin against closely related ascomycete fungal pathogens. *Mol. Plant Microbe Interact.* 32 1649–1664. 10.1094/MPMI-08-19-0224-R 31425003

[B143] Li X. S. Reddy M. S. Baev D. Edgerton M. (2003). *Candida albicans* Ssa1/2p is the cell envelope binding protein for human salivary histatin 5. *J. Biol. Chem.* 278 28553–28561. 10.1074/jbc.M300680200 12761219

[B144] Liu L. Roberts A. A. Ganz T. (2003). By IL-1 signaling, monocyte-derived cells dramatically enhance the epidermal antimicrobial response to lipopolysaccharide. *J. Immunol.* 170 575–580. 10.4049/jimmunol.170.1.575 12496445

[B145] Liu Y. Levine B. (2015). Autosis and autophagic cell death: the dark side of autophagy. *Cell Death Differ*. 22 367–376. 10.1038/cdd.2014.143 25257169 PMC4326571

[B146] Lobo D. S. Pereira I. B. Fragel-Madeira L. De Medeiros L. N. Cabral L. M. Faria J. (2007). Antifungal *Pisum sativum* defensin 1 interacts with *Neurospora crassa* cyclin F related to the cell cycle. *Biochemistry* 46 987–996. 10.1021/bi061441j 17240982

[B147] Lockhart S. R. Etienne K. A. Vallabhaneni S. Farooqi J. Chowdhary A. Govender N. P. (2017). Simultaneous emergence of multidrug-resistant *Candida auris* on 3 continents confirmed by whole-genome sequencing and epidemiological analyses. *Clin. Infect. Dis.* 64 134–140. 10.1093/cid/ciw691 27988485 PMC5215215

[B148] López-García B. Lee P. H. A. Yamasaki K. Gallo R. L. (2005). Anti-fungal activity of cathelicidins and their potential role in *Candida albicans* skin infection. *J. Invest. Dermatol.* 125 108–115. 10.1111/j.0022-202X.2005.23713.x 15982310

[B149] Manners J. M. Penninckx I. A. M. A. Vermaere K. Kazan K. Brown R. L. Morgan A. (1998). The promoter of the plant defensin gene PDF1.2 from *Arabidopsis* is systemically activated by fungal pathogens and responds to methyl jasmonate but not to salicylic acid. *Plant Mol. Biol.* 38 1071–1080. 10.1023/A:1006070413843 9869413

[B150] Martinez Del, Pozo A. Lacadena V. Mancheno J. M. Olmo N. Onaderra M. (2002). The antifungal protein AFP of *Aspergillus giganteus* is an oligonucleotide/oligosaccharide binding (OB) fold-containing protein that produces condensation of DNA. *J. Biol. Chem.* 277 46179–46183. 10.1074/jbc.M207472200 12351633

[B151] Martínez-Muñoz G. A. Kane P. (2008). Vacuolar and plasma membrane proton pumps collaborate to achieve cytosolic pH homeostasis in yeast. *J. Biol. Chem.* 283 7743–7743. 10.1074/jbc.M710470200 18502746 PMC2459297

[B152] McCaslin T. G. Pagba C. V. Yohannan J. Barry B. A. (2019). Specific metallo-protein interactions and antimicrobial activity in histatin 5, an intrinsically disordered salivary peptide. *Sci. Rep.* 9:17303. 10.1038/s41598-019-52676-7 31754129 PMC6872563

[B153] McGregor D. P. (2008). Discovering and improving novel peptide therapeutics. *Curr. Opin. Pharmacol.* 8 616–619. 10.1016/j.coph.2008.06.002 18602024

[B154] Mello ÉO. Ribeiro S. F. F. Carvalho A. O. Santos I. S. Da Cunha M. Santa-Catarina C. (2011). Antifungal activity of PvD1 defensin involves plasma membrane permeabilization, inhibition of medium acidification, and induction of ROS in fungi cells. *Curr. Microbiol.* 62 1209–1217. 10.1007/s00284-010-9847-3 21170711

[B155] Mello ÉO. Santos I. S. Carvalho A. O. Souza L. S. Souza-Filho G. A. Nascimento V. V. (2014). Functional expression and activity of the recombinant antifungal defensin PvD1r from *Phaseolus vulgaris* L. (common bean) seeds. *BMC Biochem.* 15:7. 10.1186/1471-2091-15-7 24690228 PMC3996258

[B156] Mercier T. Guldentops E. Lagrou K. Maertens J. (2018). Galactomannan, a surrogate marker for outcome in invasive aspergillosis: finally coming of age. *Front. Microbiol*. 9:661. 10.3389/fmicb.2018.00661 29670608 PMC5893815

[B157] Molino D. Nascimbeni A. C. Giordano F. Codogno P. Morel E. (2017). ER-driven membrane contact sites: evolutionary conserved machineries for stress response and autophagy regulation? *Commun. Integr. Biol.* 10:e1401699. 10.1080/19420889.2017.1401699 29259731 PMC5731517

[B158] Mookherjee N. Anderson M. A. Haagsman H. P. Davidson D. J. (2020). Antimicrobial host defence peptides: functions and clinical potential. *Nat. Rev. Drug Discov.* 19 311–332. 10.1038/s41573-019-0058-8 32107480

[B159] Moreno A. B. Martínez Del, Pozo A. San Segundo B. (2006). Biotechnologically relevant enzymes and proteins. Antifungal mechanism of the *Aspergillus giganteus* AFP against the rice blast fungus *Magnaporthe grisea*. *Appl. Microbiol. Biotechnol.* 72 883–895. 10.1007/s00253-006-0362-1 16557374

[B160] Morton C. O. Hayes A. Wilson M. Rash B. M. Oliver S. G. Coote P. (2007). Global phenotype screening and transcript analysis outlines the inhibitory mode(s) of action of two amphibian-derived, alpha-helical, cationic peptides on *Saccharomyces cerevisiae*. *Antimicrob. Agents Chemother.* 51 3948–3959. 10.1128/AAC.01007-07 17846143 PMC2151447

[B161] Muñoz A. Chu M. Marris P. I. Sagaram U. S. Kaur J. Shah D. M. (2014). Specific domains of plant defensins differentially disrupt colony initiation, cell fusion and calcium homeostasis in *Neurospora crassa*. *Mol. Microbiol.* 92 1357–1374. 10.1111/mmi.12634 24773060

[B162] Muñoz A. López-García B. Marcos J. F. (2007). Comparative study of antimicrobial peptides to control citrus postharvest decay caused by *Penicillium digitatum*. *J. Agric. Food Chem.* 55 8170–8176. 10.1021/jf0718143 17867640

[B163] Narayan K. Lemmon M. A. (2006). Determining selectivity of phosphoinositide-binding domains. *Methods* 39 122–133. 10.1016/j.ymeth.2006.05.006 16829131 PMC3786563

[B164] Nascimbeni A. C. Giordano F. Dupont N. Grasso D. Vaccaro M. I. Codogno P. (2017). ER-plasma membrane contact sites contribute to autophagosome biogenesis by regulation of local PI3P synthesis. *EMBO J.* 36 2018–2033. 10.15252/embj.201797006 28550152 PMC5509996

[B165] Noble S. M. French S. Kohn L. A. Chen V. Johnson A. D. (2010). Systematic screens of a *Candida albicans* homozygous deletion library decouple morphogenetic switching and pathogenicity. *Nat. Gen.* 42 590–598. 10.1038/ng.605 20543849 PMC2893244

[B166] Norris H. L. Kumar R. Ong C. Y. Xu D. Edgerton M. (2020). Zinc binding by histatin 5 promotes fungicidal membrane disruption in *C. albicans* and *C. glabrata*. *J. Fungi* 6 1–16. 10.3390/jof6030124 32751915 PMC7559477

[B167] Oberparleiter C. Kaiserer L. Haas H. Ladurner P. Andratsch M. Marx F. (2003). Active internalization of the *Penicillium chrysogenum* antifungal protein PAF in sensitive Aspergilli. *Antimicrob. Agents Chemother.* 47 3598–3601. 10.1128/AAC.47.11.3598-3601.2003 14576124 PMC253792

[B168] Ochiai A. Ogawa K. Fukuda M. Ohori M. Kanaoka T. Tanaka T. (2018). Rice defensin OsAFP1 is a new drug candidate against human pathogenic fungi. *Sci. Rep.* 8:11434. 10.1038/s41598-018-29715-w 30061724 PMC6065317

[B169] Ochiai A. Ogawa K. Fukuda M. Suzuki M. Ito K. Tanaka T. (2020). Crystal structure of rice defensin OsAFP1 and molecular insight into lipid-binding. *J. Biosci. Bioeng.* 130 6–13. 10.1016/j.jbiosc.2020.02.011 32192842

[B170] O’Neil D. A. Porter E. M. Elewaut D. Anderson G. M. Eckmann L. Ganz T. (1999). Expression and regulation of the human beta-defensins hBD-1 and hBD-2 in intestinal epithelium. *J. Immunol.* 163 6718–6724. 10586069

[B171] Ordonez S. R. Amarullah I. H. Wubbolts R. W. Veldhuizen E. J. A. Haagsman H. P. (2014). Fungicidal mechanisms of cathelicidins LL-37 and CATH-2 revealed by live-cell imaging. *Antimicr. Agents Chemother.* 58 2240–2248. 10.1128/AAC.01670-13 24492359 PMC4023799

[B172] Osborn R. W. De Samblanx G. W. Thevissen K. Goderis I. Torrekens S. Van Leuven F. (1995). Isolation and characterisation of plant defensins from seeds of *Asteraceae*, *Fabaceae*, *Hippocastanaceae* and *Saxifragaceae*. *FEBS Lett.* 368 257–262. 10.1016/0014-5793(95)00666-W 7628617

[B173] Oudhoff M. J. Bolscher J. G. M. Nazmi K. Kalay H. Van’t Hof W. Amerongen A. V. N. (2008). Histatins are the major wound-closure stimulating factors in human saliva as identified in a cell culture assay. *FASEB J.* 22 3805–3812. 10.1096/fj.08-112003 18650243

[B174] Paege N. Warnecke D. Zäuner S. Hagen S. Rodrigues A. Baumann B. (2019). Species-specific differences in the susceptibility of fungi to the antifungal protein AFP depend on C-3 saturation of glycosylceramides. *mSphere* 4:e00741-19. 10.1128/mSphere.00741-19 31826973 PMC6908424

[B175] Parisi K. Doyle S. R. Lee E. Lowe R. G. T. van der Weerden N. L. Anderson M. A. (2019a). Screening the *Saccharomyces cerevisiae* nonessential gene deletion library reveals diverse mechanisms of action for antifungal plant defensins. *Antimicr. Agents Chemother.* 63:e01097-19. 10.1128/AAC.01097-19 31451498 PMC6811411

[B176] Parisi K. Shafee T. M. A. Quimbar P. van der Weerden N. L. Bleackley M. R. Anderson M. A. (2019b). The evolution, function and mechanisms of action for plant defensins. *Semin. Cell Dev. Biol.* 88 107–118. 10.1016/j.semcdb.2018.02.004 29432955

[B177] Park C. Lee D. G. (2010). Melittin induces apoptotic features in *Candida albicans*. *Biochem. Biophys. Res. Commun.* 394 170–172. 10.1016/j.bbrc.2010.02.138 20188067

[B178] Parvy J. P. Yu Y. Dostalova A. Kondo S. Kurjan A. Bulet P. (2019). The antimicrobial peptide defensin cooperates with tumour necrosis factor to drive tumour cell death in *Drosophila*. *eLife* 8:e45061. 10.7554/eLife.45061 31358113 PMC6667213

[B179] Pathirana R. U. Friedman J. Norris H. L. Salvatori O. McCall A. D. Kay J. (2018). Fluconazole-resistant *Candida auris* is susceptible to salivary histatin 5 killing and to intrinsic host defenses. *Antimicr. Agents Chemother.* 62:e01872-17. 10.1128/AAC.01872-17 29158282 PMC5786754

[B180] Paulsen V. S. Blencke H. M. Benincasa M. Haug T. Eksteen J. J. Styrvold O. B. (2013). Structure-activity relationships of the antimicrobial peptide arasin 1 - and mode of action studies of the N-terminal, proline-rich region. *PLoS One* 8:e53326. 10.1371/journal.pone.0053326 23326415 PMC3543460

[B181] Payne J. A. E. Bleackley M. R. Lee T. H. Shafee T. M. A. Poon I. K. H. Hulett M. D. (2016). The plant defensin NaD1 introduces membrane disorder through a specific interaction with the lipid, phosphatidylinositol 4,5 bisphosphate. *Biochim. Biophys. Acta Biomembr.* 1858 1099–1109. 10.1016/j.bbamem.2016.02.016 26896695

[B182] Penninckx I. A. Eggermont K. Terras F. R. Thomma B. P. De Samblanx G. W. Buchala A. (1996). Pathogen-induced systemic activation of a plant defensin gene in *Arabidopsis* follows a salicylic acid-independent pathway. *Plant Cell* 8 2309–2323. 10.1105/tpc.8.12.2309 8989885 PMC161354

[B183] Phan T. K. Lay F. T. Poon I. K. H. Hinds M. G. Kvansakul M. Hulett M. D. (2016). Human β-defensin 3 contains an oncolytic motif that binds PI(4,5)P_2_ to mediate tumour cell permeabilisation. *Oncotarget* 7 2054–2069. 10.18632/oncotarget.6520 26657293 PMC4811302

[B184] Phillips M. J. Voeltz G. K. (2016). Structure and function of ER membrane contact sites with other organelles. *Nat. Rev. Mol. Cell Biol.* 17 69–82. 10.1038/nrm.2015.8 26627931 PMC5117888

[B185] Poon I. K. H. Baxter A. A. Lay F. T. Mills G. D. Adda C. G. Payne J. A. E. (2014). Phosphoinositide-mediated oligomerization of a defensin induces cell lysis. *eLife* 3:e01808. 10.7554/eLife.01808 24692446 PMC3968744

[B186] Promore Pharma (2021). *Ropocamptide – Healing of Chronic Wounds.* Available online at: https://www.promorepharma.com/en/ll-37-healing-of-chronic-wounds/ (accessed on 24 February 2021)

[B187] Puri S. Edgerton M. (2014). How does it kill?: understanding the candidacidal mechanism of salivary histatin 5. *Eukaryot. Cell* 13 958–964. 10.1128/EC.00095-14 24951439 PMC4135785

[B188] Ramamoorthy V. Cahoon E. B. Thokala M. Minto R. E. Shah D. M. (2007). Glucosylceramide synthase is essential for alfalfa defensin-mediated growth inhibition but not for pathogenicity of *Fusarium graminearum*. *Mol. Microbiol.* 66 771–786. 10.1111/j.1365-2958.2007.05955.x 17908205

[B189] Redza-Dutordoir M. Averill-Bates D. A. (2016). Activation of apoptosis signalling pathways by reactive oxygen species. *Biochim. Biophys. Acta Mol. Cell Res.* 1863 2977–2992. 10.1016/j.bbamcr.2016.09.012 27646922

[B190] Rego A. Trindade D. Chaves S. R. Manon S. Costa V. Sousa M. J. (2014). The yeast model system as a tool towards the understanding of apoptosis regulation by sphingolipids. *FEMS Yeast Res.* 14 160–178. 10.1111/1567-1364.12096 24103214

[B191] Revie N. M. Iyer K. R. Robbins N. Cowen L. E. (2018). Antifungal drug resistance: evolution, mechanisms and impact. *Curr. Opin. Microb*. 45 70–76. 10.1016/j.mib.2018.02.005 29547801 PMC6135714

[B192] Rodrigues M. L. Travassos L. R. Miranda K. R. Franzen A. J. Rozental S. de Souza W. (2000). Human antibodies against a purified glucosylceramide from *Cryptococcus neoformans* inhibit cell budding and fungal growth. *Infect. Immun*. 68 7049–7060. 10.1128/IAI.68.12.7049-7060.2000 11083830 PMC97815

[B193] Sagaram U. S. El-Mounadi K. Buchko G. W. Berg H. R. Kaur J. Pandurangi R. S. (2013). Structural and functional studies of a phosphatidic acid-binding antifungal plant defensin MtDef4: identification of an RGFRRR motif governing fungal cell entry. *PLoS One* 8:e82485. 10.1371/journal.pone.0082485 24324798 PMC3853197

[B194] Sagaram U. S. Pandurangi R. Kaur J. Smith T. J. Shah D. M. (2011). Structure-activity determinants in antifungal plant defensins Msdef1 and Mtdef4 with different modes of action against *Fusarium graminearum*. *PLoS One* 6:e18550. 10.1371/journal.pone.0018550 21533249 PMC3076432

[B195] Saito K. Takakuwa N. Ohnishi M. Oda Y. (2006). Presence of glucosylceramide in yeast and its relation to alkali tolerance of yeast. *Appl. Microbiol. Biotechnol.* 71 515–521. 10.1007/s00253-005-0187-3 16228202

[B196] Sampaio-Marques B. Burhans W. C. Ludovico P. (2019). Yeast at the forefront of research on ageing and age-related diseases. *Prog. Mol. Subcell. Biol*. 58 217–242. 10.1007/978-3-030-13035-0_9 30911895

[B197] Sardar A. H. Das S. Agnihorti S. Kumar M. Ghosh A. K. Abhishek K. (2013). Spinigerin induces apoptotic like cell death in a caspase independent manner in *Leishmania donovani*. *Exp. Parasitol.* 135 715–725. 10.1016/j.exppara.2013.10.011 24184774

[B198] Sathoff A. E. Velivelli S. Shah D. M. Samac D. A. (2019). Plant defensin peptides have antifungal and antibacterial activity against human and plant pathogens. *Phytopathology* 109 402–408. 10.1094/PHYTO-09-18-0331-R 30252607

[B199] Scarsini M. Tomasinsig L. Arzese A. D’Este F. Oro D. Skerlavaj B. (2015). Antifungal activity of cathelicidin peptides against planktonic and biofilm cultures of *Candida* species isolated from vaginal infections. *Peptides* 71 211–221. 10.1016/j.peptides.2015.07.023 26238597

[B200] Schittek B. Hipfel R. Sauer B. Bauer J. Kalbacher H. Stevanovic S. (2001). Dermcidin: a novel human antibiotic peptide secreted by sweat glands. *Nat. Immunol.* 2 1133–1137. 10.1038/ni732 11694882

[B201] Scorrano L. De Matteis M. A. Emr S. Giordano F. Hajnóczky G. Kornmann B. (2019). Coming together to define membrane contact sites. *Nat. Commun.* 10:1287. 10.1038/s41467-019-09253-3 30894536 PMC6427007

[B202] Shah P. Hsiao F. S. H. Ho Y. H. Chen C. S. (2016). The proteome targets of intracellular targeting antimicrobial peptides. *Proteomics* 16 1225–1237. 10.1002/pmic.201500380 26648572

[B203] Shah P. Wu W. S. Chen C. S. (2019). Systematical analysis of the protein targets of lactoferricin B and histatin 5 using yeast proteome microarrays. *Int. J. Mol. Sci. Art.* 20 4218–4238. 10.3390/ijms20174218 31466342 PMC6747642

[B204] Shin S. Y. Kang S. W. Lee D. G. Eom S. H. Song W. K. Kim J. I. (2000). CRAMP analogues having potent antibiotic activity against bacterial, fungal, and tumor cells without hemolytic activity. *Biochem. Biophys. Res. Commun.* 275 904–909. 10.1006/bbrc.2000.3269 10973820

[B205] Silvestro L. Weiser J. N. Axelsen P. H. (2000). Antibacterial and antimembrane activities of cecropin A in *Escherichia coli*. *Antimicr. Agents Chemother.* 44 602–607. 10.1128/AAC.44.3.602-607.2000 10681325 PMC89733

[B206] Simon A. Kullberg B. J. Tripet B. Boerman O. C. Zeeuwen P. van der Ven-Jongekrijg J. (2008). Drosomycin-like defensin, a human homologue of *Drosophila melanogaster* drosomycin with antifungal activity. *Antimicrob. Agents Chemother.* 52 1407–1412. 10.1128/AAC.00155-07 18212107 PMC2292511

[B207] Slazak B. Kapusta M. Strömstedt A. A. Słomka A. Krychowiak M. Shariatgorji M. (2018). How does the sweet violet (*Viola odorata* L.) fight pathogens and pests – cyclotides as a comprehensive plant host defense system. *Front. Plant Sci.* 9:1296. 10.3389/fpls.2018.01296 30254654 PMC6141879

[B208] Soares J. R. de Melo E. J. T. Cunha M. Fernandes K. V. S. Taveira G. B. Pereira L. S. (2017). Interaction between the plant ApDef1 defensin and *Saccharomyces cerevisiae* results in yeast death through a cell cycle- and caspase-dependent process occurring via uncontrolled oxidative stress. *Biochim. Biophys. Acta Gen. Subj.* 1861 3429–3443. 10.1016/j.bbagen.2016.09.005 27614033

[B209] Sørensen O. E. Cowland J. B. Theilgaard-Mönch K. Liu L. Ganz T. Borregaard N. (2003). Wound healing and expression of antimicrobial peptides/polypeptides in human keratinocytes, a consequence of common growth factors. *J. Immunol.* 170 5583–5589. 10.4049/jimmunol.170.11.5583 12759437

[B210] Spitzer M. Robbins N. Wright G. D. (2017). Combinatorial strategies for combating invasive fungal infections. *Virulence* 8 169–185. 10.1080/21505594.2016.1196300 27268286 PMC5354157

[B211] Steiner H. Hultmark D. Engström Å Bennich H. Boman H. G. (1981). Sequence and specificity of two antibacterial proteins involved in insect immunity. *Nature* 292 246–248. 10.1038/292246a0 7019715

[B212] Struyfs C. Cools T. L. De Cremer K. Sampaio-Marques B. Ludovico P. Wasko B. M. (2020). The antifungal plant defensin HsAFP1 induces autophagy, vacuolar dysfunction and cell cycle impairment in yeast. *Biochim. Biophys. Acta Biomembr.* 1862:183255. 10.1016/j.bbamem.2020.183255 32145284 PMC7272304

[B213] Tam J. P. Lu Y. A. Yang J. L. Chiu K. W. (1999). An unusual structural motif of antimicrobial peptides containing end-to-end macrocycle and cystine-knot disulfides. *Proc. Nat. Acad. Sci. U.S.A.* 96 8913–8918. 10.1073/pnas.96.16.8913 10430870 PMC17707

[B214] Tavares P. M. Thevissen K. Cammue B. P. A. François I. E. J. A. Barreto-Bergter E. Taborda C. P. (2008). *In vitro* activity of the antifungal plant defensin RsAFP2 against *Candida* isolates and its *in vivo* efficacy in prophylactic murine models of candidiasis. *Antimicr. Agents Chemother.* 52 4522–4525. 10.1128/AAC.00448-08 18824606 PMC2592890

[B215] Teixeira V. Feio M. J. Bastos M. (2012). Role of lipids in the interaction of antimicrobial peptides with membranes. *Prog. Lipid Res.* 51 149–177. 10.1016/j.plipres.2011.12.005 22245454

[B216] Terras F. R. G. Eggermont K. Kovaleva V. Raikhel N. V. Osborn R. W. Kester A. (1995). Small cysteine-rich antifungal proteins from radish: their role in host defense. *Plant Cell* 7 573–588. 10.1105/tpc.7.5.573 7780308 PMC160805

[B217] Thapa R. K. Diep D. B. Tønnesen H. H. (2020). Topical antimicrobial peptide formulations for wound healing: current developments and future prospects. *Acta Biomater.* 103 52–67. 10.1016/j.actbio.2019.12.025 31874224

[B218] Theis T. Marx F. Salvenmoser W. Stahl U. Meyer V. (2005). New insights into the target site and mode of action of the antifungal protein of *Aspergillus giganteus*. *Res. Microbiol.* 156 47–56. 10.1016/j.resmic.2004.08.006 15636747

[B219] Theis T. Wedde M. Meyer V. Stahl U. (2003). The antifungal protein from *Aspergillus giganteus* causes membrane permeabilization. *Antimicrob. Agents Chemother.* 47 588–593. 10.1128/aac.47.2.588-593.2003 12543664 PMC151754

[B220] Thevissen K. Cammue B. P. A. Lemaire K. Winderickx J. Dickson R. C. Lester R. L. (2000a). A gene encoding a sphingolipid biosynthesis enzyme determines the sensitivity of *Saccharomyces cerevisiae* to an antifungal plant defensin from dahlia (*Dahlia merckii*). *Proc. Nat. Acad. Sci. U.S.A.* 97 9531–9536. 10.1073/pnas.160077797 10931938 PMC16899

[B221] Thevissen K. François I. E. J. A. Takemoto J. Y. Ferket K. K. A. Meert E. M. K. Cammue B. P. A. (2003). DmAMP1, an antifungal plant defensin from dahlia (*Dahlia merckii*), interacts with sphingolipids from *Saccharomyces cerevisiae*. *FEMS Microbiol. Lett*. 226 169–173. 10.1016/S0378-1097(03)00590-1 13129623

[B222] Thevissen K. Ghazi A. De Samblanx G. W. Brownlee C. Osborn R. W. Broekaert W. F. (1996). Fungal membrane responses induced by plant defensins and thionins. *J. Biol. Chem.* 271 15018–15025. 10.1074/jbc.271.25.15018 8663029

[B223] Thevissen K. Osborn R. W. Acland D. P. Broekaert W. F. (1997). Specific, high affinity binding sites for an antifungal plant defensin on *Neurospora crassa* hyphae and microsomal membranes. *J. Biol. Chem.* 272 32176–32181. 10.1074/jbc.272.51.32176 9405418

[B224] Thevissen K. Osborn R. W. Acland D. P. Broekaert W. F. (2000b). Specific binding sites for an antifungal plant defensin from dahlia (*Dahlia merckii*) on fungal cells are required for antifungal activity. *Mol. Plant Microbe Interact.* 13 54–61. 10.1094/MPMI.2000.13.1.54 10656585

[B225] Thevissen K. Tavares P. M. Xu D. Blankenship J. Vandenbosch D. Idkowiak-Baldys J. (2012). The plant defensin RsAFP2 induces cell wall stress, septin mislocalization and accumulation of ceramides in *Candida albicans*. *Mol. Microbiol.* 84 166–180. 10.1111/j.1365-2958.2012.08017.x 22384976 PMC3405362

[B226] Thevissen K. Terras F. R. G. Broekaert W. F. (1999). Permeabilization of fungal membranes by plant defensins inhibits fungal growth. *Appl. Environ. Microbiol.* 65 5451–5458. 10.1128/aem.65.12.5451-5458.1999 10584003 PMC91743

[B227] Thevissen K. Warnecke D. C. François I. E. J. A. Leipelt M. Heinz E. Ott C. (2004). Defensins from insects and plants interact with fungal glucosylceramides. *J. Biol. Chem.* 279 3900–3905. 10.1074/jbc.M311165200 14604982

[B228] Thomma B. P. H. J. Broekaert W. F. (1998). Tissue-specific expression of plant defensin genes PDF2.1 and PDF2.2 in *Arabidopsis thaliana*. *Plant Physiol. Biochem.* 36 533–537. 10.1016/S0981-9428(98)80179-4

[B229] Tian C. Gao B. Rodriguez M. D. C. Lanz-Mendoza H. Ma B. Zhu S. (2008). Gene expression, antiparasitic activity, and functional evolution of the drosomycin family. *Mol Immunol.* 45 3909–3916. 10.1016/j.molimm.2008.06.025 18657321

[B230] Tossi A. Sandri L. Giangaspero A. (2000). Amphipathic, α-helical antimicrobial peptides. *Biopolymers* 55 4–30. 10.1002/1097-0282200055:1<4::aid-bip30<3.0.co;2-m 10931439

[B231] Tóth L. Kele Z. Borics A. Nagy L. G. Váradi G. Virágh M. (2016). NFAP2, a novel cysteine-rich anti-yeast protein from *Neosartorya fischeri* NRRL 181: isolation and characterization. *AMB Express* 6:75. 10.1186/s13568-016-0250-8 27637945 PMC5025423

[B232] Tóth L. Váradi G. Borics A. Batta G. Kele Z. Vendrinszky Á, et al. (2018). Anti-candidal activity and functional mapping of recombinant and synthetic *Neosartorya fischeri* antifungal protein 2 (NFAP2). *Front. Microbiol.* 9:393. 10.3389/fmicb.2018.00393 29563903 PMC5845869

[B233] Tsai P. W. Yang C. Y. Chang H. T. Lan C. Y. (2011). Human antimicrobial peptide LL-37 inhibits adhesion of *Candida albicans* by interacting with yeast cell-wall carbohydrates. *PLoS One* 6:17755. 10.1371/journal.pone.0017755 21448240 PMC3056723

[B234] Turner J. Cho Y. Dinh N. N. Waring A. J. Lehrer R. I. (1998). Activities of LL-37, a cathelin-associated antimicrobial peptide of human neutrophils. *Antimicrob. Agents Chemother.* 42 2206–2214. 10.1128/AAC.42.9.2206 9736536 PMC105778

[B235] van der Weerden N. L. Bleackley M. R. Anderson M. A. (2013). Properties and mechanisms of action of naturally occurring antifungal peptides. *Cell Mol. Life Sci.* 70 3545–3570. 10.1007/s00018-013-1260-1 23381653 PMC11114075

[B236] van der Weerden N. L. Hancock R. E. W. Anderson M. A. (2010). Permeabilization of fungal hyphae by the plant defensin NaD1 occurs through a cell wall-dependent process. *J. Biol. Chem.* 285 37513–37520. 10.1074/jbc.M110.134882 20861017 PMC2988356

[B237] van der Weerden N. L. Lay F. T. Anderson M. A. (2008). The plant defensin, NaD1, enters the cytoplasm of *Fusarium oxysporum* hyphae. *J. Biol. Chem.* 283 14445–14452. 10.1074/jbc.M709867200 18339623

[B238] Vandecandelaere I. Coenye T. (2015). Microbial composition and antibiotic resistance of biofilms recovered from endotracheal tubes of mechanically ventilated patients. *Adv. Exp. Med. Biol.* 830 137–155. 10.1007/978-3-319-11038-7_9 25366226

[B239] Vieira M. E. B. Vasconcelos I. M. Machado O. L. T. Gomes V. M. Carvalho A. O. (2015). Isolation, characterization and mechanism of action of an antimicrobial peptide from *Lecythis pisonis* seeds with inhibitory activity against *Candida albicans*. *Acta Biochim. Biophys. Sin.* 47 716–729. 10.1093/abbs/gmv071 26245301

[B240] Viejo-Díaz M. Andrés M. T. Fierro J. F. (2004). Modulation of *in vitro* fungicidal activity of human lactoferrin against *Candida albicans* by extracellular cation concentration and target cell metabolic activity. *Antimicr. Agents Chemother.* 48 1242–1248. 10.1128/AAC.48.4.1242-1248.2004 15047526 PMC375254

[B241] von Deuster C. I. E. Knecht V. (2011). Competing interactions for antimicrobial selectivity based on charge complementarity. *Biochim. Biophys. Acta* 1808 2867–2876. 10.1016/j.bbamem.2011.08.005 21893025

[B242] Vriens K. (2015). *Mechanisms of Action of Antifungal Agents: Antibiofilm and Ion Channel Inhibitory Properties of Plant Defensins, and Single Cell Analysis of Amphotericin B-Induced Oxidative Stress.* Dissertation. Leuven: University of Leuven.

[B243] Vriens K. Cools T. L. Harvey P. J. Craik D. J. Braem A. Vleugels J. (2016a). The radish defensins RsAFP1 and RsAFP2 act synergistically with caspofungin against *Candida albicans* biofilms. *Peptides* 75 71–79. 10.1016/j.peptides.2015.11.001 26592804

[B244] Vriens K. Cools T. L. Harvey P. J. Craik D. J. Spincemaille P. Cassiman D. (2015). Synergistic activity of the plant defensin HsAFP1 and caspofungin against *Candida albicans* biofilms and planktonic cultures. *PLoS One* 10:e0132701. 10.1371/journal.pone.0132701 26248029 PMC4527839

[B245] Vriens K. Kumar P. T. Struyfs C. Cools T. L. Spincemaille P. Kokalj T. (2017). Increasing the fungicidal action of amphotericin B by inhibiting the nitric oxide-dependent tolerance pathway. *Oxid. Med. Cell. Longev.* 2017 4064628. 10.1155/2017/4064628 29129987 PMC5654257

[B246] Vriens K. Peigneur S. De Coninck B. Tytgat J. Cammue B. P. A. Thevissen K. (2016b). The antifungal plant defensin AtPDF2.3 from *Arabidopsis thaliana* blocks potassium channels. *Sci. Rep.* 6:32121. 10.1038/srep32121 27573545 PMC5004176

[B247] Vylkova S. Li X. S. Berner J. C. Edgerton M. (2006). Distinct antifungal mechanisms: β-defensins require *Candida albicans* Ssa1 protein, while Trk1p mediates activity of cysteine-free cationic peptides. *Antimicrob. Agents Chemother.* 50 324–331. 10.1128/AAC.50.1.324-331.2006 16377704 PMC1346820

[B248] Vylkova S. Nayyar N. Li W. Edgerton M. (2007). Human β-defensins kill *Candida albicans* in an energy-dependent and salt-sensitive manner without causing membrane disruption. *Antimicrob. Agents Chemother.* 51 154–161. 10.1128/AAC.00478-06 17074797 PMC1797696

[B249] Wachinger M. Kleinschmidt A. Winder D. Von Pechmann N. Ludvigsen A. Neumann M. (1998). Antimicrobial peptides melittin and cecropin inhibit replication of human immunodeficiency virus 1 by suppressing viral gene expression. *J. Gen. Virol.* 79 731–740. 10.1099/0022-1317-79-4-731 9568968

[B250] Wang G. Li X. Wang Z. (2016). APD3: the antimicrobial peptide database as a tool for research and education. *Nucleic Acids Res.* 44 1087–1093. 10.1093/nar/gkv1278 26602694 PMC4702905

[B251] Wang J. Xia X. M. Wang H. Y. Li P. P. Wang K. Y. (2013). Inhibitory effect of lactoferrin against gray mould on tomato plants caused by *Botrytis cinerea* and possible mechanisms of action. *Int. J. Food Microbiol.* 161 151–157. 10.1016/j.ijfoodmicro.2012.11.025 23333340

[B252] Wilmes M. Cammue B. P. A. Sahl H.-G. Thevissen K. (2011). Antibiotic activities of host defense peptides : more to it than lipid bilayer perturbation. *Nat. Prod. Rep*. 28 1350–1358. 10.1039/c1np00022e 21617811

[B253] World Health Organization [WHO] (2020). *Antimicrobial Resistance.* Geneva: World Health Organization.

[B254] Yount N. Y. Kupferwasser D. Spisni A. Dutz S. M. Ramjan Z. H. Sharma S. (2009). Selective reciprocity in antimicrobial activity versus cytotoxicity of hBD-2 and crotamine. *Proc. Nat. Acad. Sci. U.S.A.* 106 14972–14977. 10.1073/pnas.0904465106 19706485 PMC2736466

[B255] Yun J. E. Lee D. G. (2016). Cecropin A-induced apoptosis is regulated by ion balance and glutathione antioxidant system in *Candida albicans*. *IUBMB Life* 68 652–662. 10.1002/iub.1527 27338801

[B256] Zasloff M. (1987). Magainins, a class of antimicrobial peptides from *Xenopus* skin: isolation, characterization of two active forms, and partial cDNA sequence of a precursor. *Proc. Nat. Acad. Sci. U.S.A.* 84 5449–5453. 10.1073/pnas.84.15.5449 3299384 PMC298875

[B257] Zasloff M. (2002). Antimicrobial peptides of multicellular organisms. *Nature* 415 389–395. 10.1038/415389a 11807545

