# Supplementary material for: Membrane-Interacting Antifungal Peptides
Source: Front Cell Dev Biol. 2021 Apr 12;9:649875. doi: 10.3389/fcell.2021.649875 (PMC8074791; doi:10.3389/fcell.2021.649875)
Supplement: Supplementary file 1 [file Table_1.docx]

| **Table 1.** Summary of the main characteristics of various AMPs, described in this review. | | | | | | | |
| --- | --- | --- | --- | --- | --- | --- | --- |
| **Origin** | **Antifungal peptide** | **Species of origin** | **Cell surface target** | **Cellular uptake** | **Mechanism of antifungal action** | **Susceptible fungal pathogens** | **Reference(s)** |
| **Fungal** | AFP | *Aspergillus giganteus* | Chitin, GlcCer | Yes | DNA condensation  Membrane permeabilization | *Aspergillus awamorii, A. nidulans, A. niger, A. oryzae, Fusarium bulbigenum, F. equiseti, F. lactis, F. lini, F. moniliforme, F. oxysporum, F. poae, F. proliferatum, F. solani, F. sporotrichoides, F. vasinfectum, Magnaporthe grisea* | (Martinez et al. 2002; Theis et al. 2003, 2005; Moreno et al. 2006; Hagen et al. 2007; Paege et al. 2019) |
|  | NFAP | *Neosartorya fischeri* | Unknown | Yes | Energy-dependent uptake in *N. crassa*  Damage and dysfunction of cell wall  Destruction of chitin filaments  Accumulation of nuclei at broken hyphal tips  Programmed cell death | *Aspergillus nidulans, A. niger, Neurospora crassa, Rhizomucor miehei* | (Kovács et al. 2011; Galgóczy et al. 2013; Hajdu et al. 2019) |
|  | NFAP2 | *Neosartorya fischeri* | Unknown | Unknown | Membrane permeabilization | *Candida albicans, C. auris, C. glabrata, C. guilliermondii, C. krusei, C. lusitaniae, C. parapsilosis, C. tropicalis, Saccharomyces cerevisiae, Schizosaccharomyces pombe* | (Tóth et al. 2016, 2018; Kovács et al. 2019, 2021) |

| **Table 1.** *(continued)* | | | | | | | |
| --- | --- | --- | --- | --- | --- | --- | --- |
| **Origin** | **Antifungal peptide** | **Species of origin** | **Cell surface target** | **Cellular uptake** | **Mechanism of antifungal action** | **Susceptible fungal pathogens** | **Reference(s)** |
|  | PAF | *Penicillium chrysogenum* | GlcCer | Yes | Endocytosis-mediated uptake  ROS production  K^+^ efflux  Ca^2+^ influx  Reduction of conidial germination and hyphal extension rates  Crippled and distorted hyphae and atypical branching  Programmed cell death | *Aspergillus flavus, A.* *fumigatus, A. giganteus, A. nidulans, A. niger, Botrytis cinerea, Cochliobolus carbonum, Fusarium oxysporum, Gliocladium roseum, Neurospora crassa, Penicillium chrysogenum, Trichoderma koningii* | (Kaiserer et al. 2003; Oberparleiter et al. 2003; Leiter et al. 2005; Binder et al. 2010, 2015) |
|  | PAFB | *Penicillium chrysogenum* | Unknown | Yes | Membrane permeabilization | *Aspergillus fumigatus, A. niger, A. terreus, Candida albicans, C. glabrata, C. krusei, C. parapsilosis, Neosartorya fischeri, Neurospora crassa, Penicillium chrysogenum, Saccharomyces cerevisiae, Trichophyton mentagrophytes,* T. rubrum | (Huber et al. 2018, 2019a, 2019b, 2020) |

| **Table 1.** *(continued)* | | | | | | | |
| --- | --- | --- | --- | --- | --- | --- | --- |
| **Origin** | **Antifungal peptide** | **Species of origin** | **Cell surface target** | **Cellular uptake** | **Mechanism of antifungal action** | **Susceptible fungal pathogens** | **Reference(s)** |
|  | PAFC | *Penicillium chrysogenum* | Unknown | Yes | ROS production  Membrane permeabilization | *Aspergillus nidulans, Candida albicans, C. glabrata, C. guilliermondii, C. krusei, C. parapsilosis, Microsporum gypseum, Neurospora crassa, Penicillium chrysogenum, Trichophyton rubrum* | (Holzknecht et al. 2020; Czajlik et al. 2021) |
| **Plant** | ApDef1 | *Adenanthera pavonina* | Unknown | Unknown | Cell cycle dysfunction  ROS production  Programmed cell death | *Saccharomyces cerevisiae* | (Soares et al. 2017) |
|  | AtPDF2.3 | *Arabidopsis thaliana* | M(IP)_2_C | Unknown |  | *Botrytis cinerea, Fusarium culmorum, F. graminearum, F. oxysporum, Saccharomyces cerevisiae, Verticillium dahlia* | (Vriens 2015; Vriens et al. 2016a) |
|  | CyO2 | *Viola odorata* | PE | Unknown | Membrane permeabilization | *Alternaria alternate, Botrytis cinerea, Colletotrichum utrechtense, Fusarium culmorum, F. graminearum, F. oxysporum* | (Burman et al. 2011; Henriques et al. 2012; Slazak et al. 2018) |

| **Table 1.** *(continued)* | | | | | | | |
| --- | --- | --- | --- | --- | --- | --- | --- |
| **Origin** | **Antifungal peptide** | **Species of origin** | **Cell surface target** | **Cellular uptake** | **Mechanism of antifungal action** | **Susceptible fungal pathogens** | **Reference(s)** |
|  | DmAMP1 | *Dahlia merckii* | M(IP)_2_C, ergosterol | Unknown | K^+^ efflux  Ca^2+^ influx  Membrane permeabilization | *Cladosporium sphaerospermum, Fusarium culmorum, Leptosphaeria maculans, Penicillium digitatum, Saccharomyces cerevisiae, Septoria tritici* | (Osborn et al. 1995; Thevissen et al. 2000b; Thevissen et al. 2003; Aerts et al. 2006; Parisi et al. 2019b) |
|  | HsAFP1 | *Heuchera sanguinea* | PA, PIPs | Yes | Endocytosis-mediated uptake  Cell cycle impairment  Autophagy  Vacuolar dysfunction  ROS production  Mitochondrial dysfunction  Programmed cell death  Membrane permeabilization | *Botrytis cinerea, Candida albicans, C. dubliniensis, C. glabrata, C. krusei, Cladosporium sphaerospermum, Fusarium culmorum, Penicillium digitatum, Saccharomyces cerevisiae, Septoria tritici* | (Osborn et al. 1995; Aerts et al. 2011; Vriens et al. 2015; Cools et al. 2017b; Cools et al. 2017c; Struyfs et al. 2020) |
|  | LpDef1 | *Lecythis Pisonis* | Unknown | Unknown | ROS  Mitochondrial dysfunction | *Candida albicans* | (Vieira et al. 2015) |
|  | MsDef1 | *Medicago sativa* | GlcCer and PI(3,5)P_2_ | Unknown | Disruption of Ca^2+^ homeostasis  Inhibition of conidial germination  Membrane permeabilization | *Aspergillus flavus, Fusarium graminearum, F. verticillioides, Neurospora crassa* | (Allen et al. 2008; Sagaram et al. 2011, 2013; Muñoz et al. 2014) |

| **Table 1.** *(continued)* | | | | | | | |
| --- | --- | --- | --- | --- | --- | --- | --- |
| **Origin** | **Antifungal peptide** | **Species of origin** | **Cell surface target** | **Cellular uptake** | **Mechanism of antifungal action** | **Susceptible fungal pathogens** | **Reference(s)** |
|  | MtDef4 | *Medicago truncatula* | PA | Yes | Endocytosis mediated uptake  Disruption of Ca^2+^ homeostasis  Inhibition of cell fusion  Inhibition of conidial germination  Membrane permeabilization | *Aspergillus flavus, Fusarium graminearum, F.* *verticillioides, Neurospora crassa* | (Sagaram et al. 2011, 2013; Muñoz et al. 2014; El-Mounadi et al. 2016;) |
|  | NaD1 | *Nicotiana alata* | Chitin,  β-glucan, PA, PI(4,5)P_2_ | Yes | Endocytosis-mediated uptake via ESCRT independent pathway  ROS and NO production  Membrane permeabilization | *Aspergillus nidulans, A. niger, A. parasiticus, Botrytis cinerea, Candida albicans, Colletotrichum graminicola, Cryptococcus gattii, C. neoformans, Fusarium graminearum, F. oxysporum, Leptosphaeria maculans, Puccinia coronate, P. sorghi, Saccharomyces cerevisiae, Thielaviopsis basicola, Verticillium dahlia* | (Lay et al. 2003, 2012; van der Weerden et al. 2008, 2010; Poon et al. 2014; Payne et al. 2016; Bleackley et al. 2016, 2017, 2019; Hayes et al. 2018; Parisi et al. 2019a, 2019b) |
|  | NaD2 | *Nicotiana alata* | PA, PIPs | Unknown |  | *Aspergillus nidulans, Fusarium oxysporum* | (Dracatos et al. 2016) |
|  | NbD6 | *Nicotiana benthamiana* | Unknown | Yes | Vacuolar dysfunction  ROS production | *Saccharomyces cerevisiae* | (Parisi et al. 2019b) |
|  | NsD7 | *Nicotiana suaveolens* | PA and PI(4,5)P_2_ | Unknown | Membrane permeabilization |  | (Kvansakul et al. 2016) |

| **Table 1.** *(continued)* | | | | | | | |
| --- | --- | --- | --- | --- | --- | --- | --- |
| **Origin** | **Antifungal peptide** | **Species of origin** | **Cell surface target** | **Cellular uptake** | **Mechanism of antifungal action** | **Susceptible fungal pathogens** | **Reference(s)** |
|  | OefDef1.1 | *Olea europaea* | PI3P, PI5P | Yes^a^ | ROS production  Membrane permeabilization | *Botrytis cinerea, Fusarium graminearum, F. oxysporum, F. virguliforme* | (Li et al. 2019) |
|  | OsAFP1 | *Oryza sativa* | PI3P | Unknown | Programmed cell death | *Candida albicans, Saccharomyces cerevisiae* | (Ochiai et al. 2018, 2020) |
|  | Psd1 | *Pisum sativum* | GlcCer and ergosterol | Yes | Cell cycle arrest  Membrane permeabilization | *Aspergillus niger, A. versicolor, Candida albicans, Fusarium moniliforme, F. solani, Neurospora crassa* | (Almeida et al. 2000; 2002; Lobo et al. 2007; Gonçalves et al. 2017; De Medeiros et al. 2010, 2014) |
|  | Psd2 | *Pisum sativum* | GlcCer and ergosterol | Unknown |  | *Aspergillus nidulans, A. niger, A. versicolor, Candida albicans, Colletotrichum musae, Fusarium moniliforme, F. solani, Neurospora crassa* | (Almeida et al. 2000; Amaral et al. 2019) |
|  | PvD1 | *Phaseolus vulgaris* | GlcCer | Unknown | ROS and NO production  Membrane permeabilization | *Candida albicans, C. guilliermondii, C. parapsilosis, C. tropicalis, Fusarium laterithium, F. oxysporum, F. solani, Kluyveromyces marxiannus, Pichia membranifaciens, Saccharomyces cerevisiae* | (Mello et al. 2011, 2014) |

| **Table 1.** *(continued)* | | | | | | | |
| --- | --- | --- | --- | --- | --- | --- | --- |
| **Origin** | **Antifungal peptide** | **Species of origin** | **Cell surface target** | **Cellular uptake** | **Mechanism of antifungal action** | **Susceptible fungal pathogens** | **Reference(s)** |
|  | RsAFP2 | *Raphanus sativus* | GlcCer | No | ROS production  K^+^ efflux  Ca^2+^ influx  Ceramide accumulation  Metacaspase independent programmed cell death  Cell wall stress and septin mislocalization  Membrane permeabilization | *Alternaria brassicola, A. longipes, Aspergillus flavus, Botrytis cinerea, Candida albicans, C. dubliniensis, C. krusei, C. parapsilosis, C. tropicalis, Cladosporium sphaerospermum, Fusarium culmorum, F. graminearum, F. oxysporum, F. solani, Penicillium digitatum, Pichia pastoris, Phoma betae, Septoria tritiei* | (Osborn et al. 1995; Terras et al. 1995; Thevissen et al. 2004, 2012; Lay and Anderson 2005; Aerts et al. 2007; Tavares et al. 2008; Vriens et al. 2016b) |
|  | SBI6 | *Glycine max* | Unknown | Yes | Uptake could be endocytosis-mediated  Vacuolar dysfunction | *Saccharomyces cerevisiae* | (Parisi et al. 2019b) |
|  | Sd5 | *Saccharum officinarum* | GlcCer | Unknown |  | *Fusarium solani, Neurospora crassa* | (De Paula et al. 2008, 2011) |
|  | TPP3 | *Solanum lycopersicum* | PI(4,5)P_2_ | Unknown | Membrane permeabilization | *Fusarium graminearum* | (Baxter et al. 2015) |
| **Invertebrate** | Arasin 1 | *Hyas araneus* | Chitin | Unkown |  | *Botrytis cinerea, Candida albicans, Saccharomyces cerevisiae* | (Paulsen et al. 2013) |

| **Table 1.** *(continued)* | | | | | | | |
| --- | --- | --- | --- | --- | --- | --- | --- |
| **Origin** | **Antifungal peptide** | **Species of origin** | **Cell surface target** | **Cellular uptake** | **Mechanism of antifungal action** | **Susceptible fungal pathogens** | **Reference(s)** |
|  | Cecropin A | *Bombyx mori* | Unknown | Unknown | ROS production  K^+^ efflux  Ca^2+^ influx  Programmed cell death | *Candida albicans* | (Andrä, et al. 2001, Yun and Lee 2016) |
|  | Coprisin | *Copris tripartitus* | Unknown | Unknown | ROS production  Mitochondrial dysfunction  Programmed cell death | *Aspergillus flavus, A. fumigatus, A. parasiticus, Candida albicans, C. parapsilosis, Malassezia furfur, Trichosporon beigelii, Trichophyton rubrum* | (Lee et al. 2012, 2014) |
|  | Drosomycin | *Drosophila melanogaster* | Unknown | Unknown | Hyphae lysis  Inhibition of spore germination | *Alternaria brassicola, A. longipes, Ascochytu pisi, Aspergillus fumigatus, A. ustus, Botrytis cinerea, Colletotrichum gloeosporioides, Fusarium culmorum, F. oxysporum, F. solani, Geotrichum candidum, Nectria haematococca, Neurospora crassa, Saccharomyces cerevisiae* | (Fehlbaum et al. 1994; Landon et al 1997; Gao and Zhu 2008; Simon et al 2008; Tian et al. 2008; Cohen et al 2009) |

| **Table 1.** *(continued)* | | | | | | | |
| --- | --- | --- | --- | --- | --- | --- | --- |
| **Origin** | **Antifungal peptide** | **Species of origin** | **Cell surface target** | **Cellular uptake** | **Mechanism of antifungal action** | **Susceptible fungal pathogens** | **Reference(s)** |
|  | ETD151 | analogue of heliomicin | Unknown | Unknown | Dysregulation of the endocytosis pathway  Dysregulation of the spliceosome  Mitochondrial dysfunction | *Aspergillus fumigatus, Botrytis cinerea, Candida albicans, Cryptococcus neoformans, Fusarium solani, Scedosporium prolificans* | (Landon et al. 2003; Aumer et al. 2020) |
|  | Heliomicin | *Heliothis virescens* | GlcCer | Unknown |  | *Aspergillus fumigatus, Candida albicans, Fusarium culmorum, Nectria haematococca, Neurospora crassa, Pichia pastoris* | (Lamberty et al. 2001b; Ferket et al. 2003; Landon et al. 2003; Thevissen et al. 2004) |
|  | Melittin | *Apis mellifera* | Unknown | Unknown | Programmed cell death | *Candida albicans, Penicillium digitatum, Saccharomyces cerevisiae* | (Muñoz et al. 2007; Park and Lee 2010) |
|  | Scolopendin | *Scolopendra subspinipes mutilans* | Unknown | Unknown | ROS production  Ca^2+^ influx  Mitochondrial dysfunction  Programmed cell death | *Candida albicans, C. parapsilosis, Trichosporon beigelii* | (Choi et al. 2014; Lee et al. 2016, 2017) |
|  | Spinigerin | *Pseudacanth-otermes spiniger* | Unknown | Unknown |  | *Candida albicans, Fusarium culmorum, Nectria haematococca, Neurospora crassa, Trichoderma viride* | (Lamberty et al. 2001a; Sardar et al. 2013) |

| **Table 1.** *(continued)* | | | | | | | |
| --- | --- | --- | --- | --- | --- | --- | --- |
| **Origin** | **Antifungal peptide** | **Species of origin** | **Cell surface target** | **Cellular uptake** | **Mechanism of antifungal action** | **Susceptible fungal pathogens** | **Reference(s)** |
|  | Termicin | *Pseudacanth-otermes spiniger* | Unknown | Unknown |  | *Candida albicans, Cryptococcus neoformans, Fusarium culmorum, F. oxysporum, Nectria haematococca, Neurospora crassa, Saccharomyces cerevisiae, Trichoderma viride* | (Lamberty et al. 2001a) |
| **Vertebrates** | CRAMP | *Mus musculus* | Unknown | Unknown | Membrane permeabilization | *Aspergillus fumigateus, Candida albicans, Cryptococcus neoformans* | (Gallo et al. 1997; Shin et al. 2000) |
|  | hBD-1 | *Homo sapiens* | Unknown | Unknown |  | *Candida albicans, C. krusei, C. parapsilosis* | (Goldman et al. 1997, Feng et al. 2005; Vylkova et al. 2006, Krishnakumari et al. 2009) |
|  | hBD-2 | *Homo sapiens* | Ssa1p, SSa2p, PI(4,5)P_2_ | Unknown | ATP efflux  Energy dependent mechanism  Programmed cell death  Membrane permeabilization | *Candida albicans, C. krusei*  *C. parapsilosis, C. tropicalis* | (Joly et al. 2004; Feng et al. 2005; Vylkova et al. 2006, 2007; Gank et al. 2008; Yount et al. 2009; Argimón et al. 2011; Jung et al. 2013; Järvå et al. 2018b) |
|  | hBD-3 | *Homo sapiens* | Ssa1p, SSa2p, PI(4,5)P_2_ | Unknown | ATP efflux  Energy dependent mechanism | *Candida albicans, C. krusei, C. parapsilosis, C. tropicalis* | (Joly et al. 2004; Feng et al 2005; Vylkova et al. 2006, 2007; Argimón et al. 2011; Phan et al. 2016) |
| **Table 1.** *(continued)* | | | | | | | |
| **Origin** | **Antifungal peptide** | **Species of origin** | **Cell surface target** | **Cellular uptake** | **Mechanism of antifungal action** | **Susceptible fungal pathogens** | **Reference(s)** |
|  | HNP-1 | *Homo sapiens* | Ssa1p, Ssa2p | Unknown | ATP efflux | *Candida albicans, Cryptococcus neoformans* | (Ganz et al. 1985; Lehrer et al. 1988; Edgerton et al. 2000; Vylkova et al. 2006) |
|  | HNP-2 | *Homo sapiens* | Unknown | Unknown | Unknown | *Candida albicans, Cryptococcus neoformans* | (Ganz et al. 1985; Lehrer et al. 1988;) |
|  | HNP-3 | *Homo sapiens* | Unknown | Unknown | Unknown | *Cryptococcus neoformans* | (Ganz et al. 1985; Lehrer et al. 1988) |
|  | Histatin 5 | *Homo sapiens* | β-glucan, Ssa1p, Ssa2p | Yes | Endocytosis-mediated uptake and transportation through Dur3p and Dur31p  ATP efflux  ROS production  K^+^ efflux; Trk1p is required for the antifungal mode of action  Cell cycle arrest  Vacuolar expansion | *Aspergillus flavus, A. fumigatus, Candida albicans, C. auris, C. castelli, C. kefyr, C. krusei, C. parapsilosis, Cryptococcus neoformans, Kluyveromyces bacillisporus, K. delphensis, Saccharomyces cerevisiae* | (Helmerhorst et al. 1999; Koshlukova et al. 1999; Baev et al. 2002, 2004; Dong et al. 2003; Li et al. 2003; Helmerhorst et al. 2005; Jang et al. 2010; Kumar et al. 2011; Puri and Edgerton 2014; Pathirana et al. 2018; McCaslin et al. 2019; Ikonomova et al. 2020; Norris et al. 2020) |

| **Table 1.** *(continued)* | | | | | | | |
| --- | --- | --- | --- | --- | --- | --- | --- |
| **Origin** | **Antifungal peptide** | **Species of origin** | **Cell surface target** | **Cellular uptake** | **Mechanism of antifungal action** | **Susceptible fungal pathogens** | **Reference(s)** |
|  | Indolicidin | *Bos taurus* |  | Yes | Membrane permeabilization | *Aspergillus fumigatus, Candida albicans, C. dubliniensis, C. famata, C.* *humicola, C. krusei, C. lusitaniae, Cryptococcus neoformans,* Kloeckera apis, *Penicillium digitatum, Pichia carsonii,* Rhodotorula rubra, *Saccharomyces cerevisiae, Trichosporon beigelii* | (Lee et al. 2003; Benincasa et al. 2006; Muñoz et al. 2007) |
|  | Lactoferrin | *Homo sapiens* | Pma1p H^+^-ATPase |  | K^+^ efflux  ROS production  Programmed cell death  Mitochondrial dysfunction | *Aspergillus niger, Botrytis cinerea, Candida albicans, C. krusei, C. tropicalis, Saccharomyces cerevisiae, Trichoderma viride, Trichophyton mentagrophytes* | (Viejo-Díaz et al. 2004; Andrés et al. 2008; Wang et al. 2013; Acosta-Zaldívar et al. 2016) |
|  | LL-37 | *Homo sapiens* | Mannan, chitin, glucan | No | ATP efflux  Membrane disintegration | *Candida albicans, C. krusei, C.* *norvegensis, C. parapsilosis, Saccharomyces cerevisiae* | (Den Hertog et al. 2005; Tsai et al. 2011; Ordonez et al. 2014; Scarsini et al. 2015) |
|  | Magainin 2 | *Xenopus laevis* | Unknown | Unknown | Membrane permeabilization | *Candida albicans, Saccharomyces cerevisiae* | (Helmerhorst et al. 2005; Morton et al. 2007) |

| **Table 1.** *(continued)* | | | | | | | |
| --- | --- | --- | --- | --- | --- | --- | --- |
| **Origin** | **Antifungal peptide** | **Species of origin** | **Cell surface target** | **Cellular uptake** | **Mechanism of antifungal action** | **Susceptible fungal pathogens** | **Reference(s)** |
|  | Protegrin 1 | *Sus scrofa* | Unknown | Unknown | Membrane permeabilization | *Candida albicans, C. dubliniensis, C. famata, C. glabrata, C.  guillermondii, C. humicola, C. kruseii, C. lusitaniae, C. parapsilosis, C.tropicalis, Cryptococcus neoformans,* Kloeckera apis, Pichia carsonii, P. etchellsii, Rhodotorula rubra, Saccharomyces cerevisiae | (Cho et al. 1998; Benincasa et al. 2006; Do et al. 2014) |

^a^ Translocation to the cytoplasm in both germlings and conidia in *F. oxysporum* and *F. virguliforme*. However, only in germlings in *B. cinerea* and not in conidia.
